# Very long chain fatty acid-containing lipids: a decade of novel insights from the study of ELOVL4

**DOI:** 10.1016/j.jlr.2021.100030

**Published:** 2021-02-06

**Authors:** Gyening Kofi Yeboah, Ekaterina S. Lobanova, Richard S. Brush, Martin-Paul Agbaga

**Affiliations:** 1 Department of Cell Biology, University of Oklahoma Health Sciences Center, Oklahoma City, OK, USA; 2 Department of Ophthalmology Research, University of Florida, Gainesville, FL, USA; 3 Department of Ophthalmology, University of Oklahoma Health Sciences Center, Oklahoma City, OK, USA; 4 Dean A. McGee Eye Institute, Oklahoma City, OK, USA

**Keywords:** very long chain saturated fatty acids, retinal lipids, autosomal dominant, Stargardt macular dystrophy, spinocerebellar ataxia 34, erythrokeratodermia variabilis, very long chain polyunsaturated fatty acids, AA, arachidonic acid (20:4n6), AMD, age-related macular degeneration, BBS, Bardet-Biedl syndrome, ELOVL, elongation of very long-chain fatty acid, ERG, electroretinography, IS, inner segment, OS, outer segment, PC, phosphatidylcholine, POS, photoreceptor outer segment, RPE, retinal pigment epithelium, SCA34, spinocerebellar ataxia-34, STGD3, Stargardt-like macular dystrophy, VLC-SFA, very long chain saturated fatty acid, VLC-PUFA, very long chain polyunsaturated fatty acid, VLC-FA, very long chain fatty acid

## Abstract

Lipids play essential roles in maintaining cell structure and function by modulating membrane fluidity and cell signaling. The fatty acid elongase-4 (ELOVL4) protein, expressed in retina, brain, Meibomian glands, skin, testes and sperm, is an essential enzyme that mediates tissue-specific biosynthesis of both VLC-PUFA and VLC-saturated fatty acids (VLC-SFA). These fatty acids play critical roles in maintaining retina and brain function, neuroprotection, skin permeability barrier maintenance, and sperm function, among other important cellular processes. Mutations in *ELOVL4* that affect biosynthesis of these fatty acids cause several distinct tissue-specific human disorders that include blindness, age-related cerebellar atrophy and ataxia, skin disorders, early-childhood seizures, mental retardation, and mortality, which underscores the essential roles of ELOVL4 products for life. However, the mechanisms by which one tissue makes VLC-PUFA and another makes VLC-SFA, and how these fatty acids exert their important functional roles in each tissue, remain unknown. This review summarizes research over that last decade that has contributed to our current understanding of the role of ELOVL4 and its products in cellular function. In the retina, VLC-PUFA and their bioactive “Elovanoids” are essential for retinal function. In the brain, VLC-SFA are enriched in synaptic vesicles and mediate neuronal signaling by determining the rate of neurotransmitter release essential for normal neuronal function. These findings point to ELOVL4 and its products as being essential for life. Therefore, mutations and/or age-related epigenetic modifications of fatty acid biosynthetic gene activity that affect VLC-SFA and VLC-PUFA biosynthesis contribute to age-related dysfunction of ELOVL4-expressing tissues.

## Graphical abstract

**Figure:**
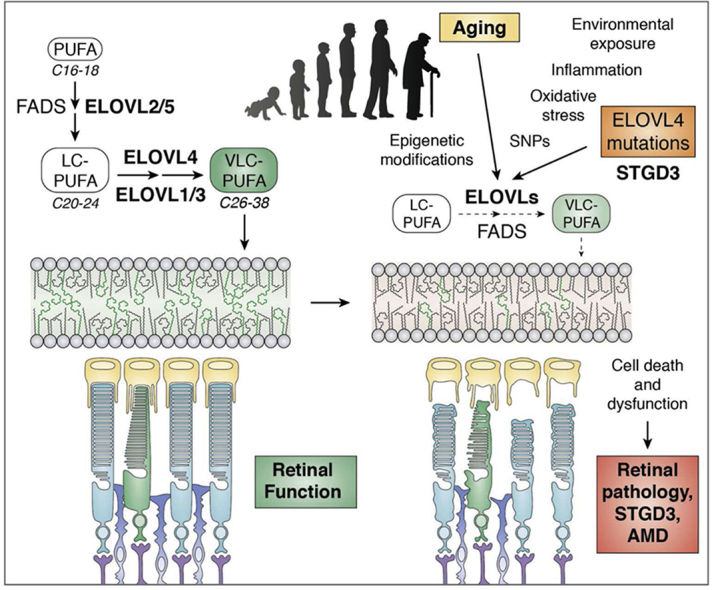

Evolving discovery of novel mutations in the *ELOngation of Very Long chain fatty acids-4* (*ELOVL4*) gene as the cause of different tissue-specific disorders in humans is providing novel insights into the role of lipids in health and disease in a number of important organs. In 2001, Zhang *et al.* (1) and Edwards *et al.* (2) independently reported a five base pair deletion (797–801delAACT) in exon 6 of the *ELOVL4* gene located on human chromosome 6q14 as the cause of early-onset macular degeneration in patients with autosomal dominant Stargardt-like macular dystrophy (STGD3). The deletion results in truncation of the last 51 amino acids, including an endoplasmic reticulum retention/retrieval signal located at the C-terminal end of the ELOVL4 protein (1, 2). The same year, Bernstein *et al.* (3) reported two different one base pair deletions separated by four nucleotides (790delT+794delT) within the same region of exon 6 of *ELOVL4*. These mutations also result in truncation of the ELOVL4 protein in a manner similar to the reported 5 bp deletion (1, 2, 3). Three years later, Maugeri *et al.* (4) reported a fourth mutation also in exon 6 of ELOVL4 (c. 810C > G; p Tyr270X) that leads to truncation of the ELOVL4 protein in a European family with STGD3 pathology. A major characteristic of these STGD3-causing mutations is early-onset loss of central vision and macular degeneration akin to age-related macular degeneration (AMD), characterized by accumulation of high levels of lipofuscin in the retinal pigment epithelium (RPE), and macular degeneration (5, 6, 7, 8, 9, 10, 11).

These discoveries generated significant interest in understanding the biological function of the ELOVL4 protein, which shares sequence homology with a group of yeast fatty acid elongase (ELO) proteins involved in fatty acid elongation (12, 13, 14, 15). A number of laboratories, including ours, carried out a series of *in vitro* (16, 17, 18, 19, 20, 21) and *in vivo* (20, 22, 23, 24, 25, 26) experiments aimed at elucidating the biological function of the ELOVL4 protein and how the mutations cause blindness in patients with STGD3. Since the retina contains high levels of PUFA, especially DHA, which represents approximately 45–50% of retinal outer segment phospholipids, initial hypotheses suggested that ELOVL4 may be involved in retinal DHA biosynthesis (1). However, our exhaustive *in vitro* studies showed that ELOVL4 is not involved in the elongation steps required for DHA biosynthesis (27). In 2008, our laboratory discovered and unequivocally reported that the wild-type (WT) ELOVL4 protein is an essential enzyme that mediates the initial rate-limiting step of the fatty acyl chain condensation reaction in very long fatty acid elongation (28). We showed that WT ELOVL4 is involved in synthesis of both very long chain polyunsaturated fatty acids (VLC-PUFA) and very long chain saturated fatty acids (VLC-SFA), which we collectively refer to as very long chain fatty acids (VLC-FA), in ELOVL4-expressing tissues (28). We subsequently showed that STGD3 mutant ELOVL4 lacks both VLC-PUFA and VLC-SFA biosynthesis and exerts a dominant negative effect on WT ELOVL4 biosynthetic activity *in vitro* (29, 30). The results from these *in vitro* studies are supported by *in vivo* studies in which homozygous expression of mutant ELOVL4 or global deletion of ELOVL4 causes neonatal lethality in mice (23, 31, 32, 33).

Apart from the initial three ELOVL4 mutations that cause STGD3 (1, 2, 3, 4), in the last decade a number of different ELOVL4 mutations that cause different tissue-specific disorders in humans have been reported (Table 1). These novel ELOVL4 mutations comprise both the heterozygous (35, 36, 37, 38, 42, 43) and homozygous forms (40, 41), with the latter leading to even more severe human disorders characterized by seizures, intellectual disability, and childhood mortality. Taken together, these results have clearly established that functional ELOVL4 and its VLC-FA products are essential for life.

**Table 1:** Different tissue-specific disorders caused by different ELOVL4 mutations in humans

Genetic Mutations	Exon	Genetic Consequence/Resultant Protein	Inheritance	Retinal Pathology	Brain Pathology	Skin Pathology	Reference
797–801 del AACTT “5 bp deletion”	6	Premature stop, truncated protein	Autosomal dominant	STGD3	None reported	None reported	(1, 2)
789 del T, 794 del T “2 bp deletion”	6	Premature stop, truncated protein	Autosomal dominant	STGD3	None reported	None reported	(3)
c.810C > G	6	p.Y270X, truncation	Autosomal dominant	STGD3	None reported	None reported	(4)
c.-90G > C	Promoter, rs62407622	Downregulation of ELOVL4 expression	Autosomal dominant	STGD3	None reported	None reported	(34)
c.-236C > T	Promoter, rs240307	Downregulation of ELOVL4 expression	Autosomal dominant	STGD3	None reported	None reported	(34)
c.504G > C	4	p.L168F	Autosomal dominant	None reported	Age-related cerebellar atrophy causing ataxia in patients with SCA34	Erythrodermia variabilis, a skin lesion disorder	(35)
c.736T > G	6	p.W246G	Autosomal dominant	None reported	Age-related cerebellar atrophy causing ataxia in patients with SCA34	None reported in human, but present in homozygous W246G knockin rats	(36)
c.539A > C	4	p.G180P	Autosomal dominant	None reported	Age-related cerebellar atrophy causing ataxia in patients with SCA34	Erythrodermia variabilis, a skin lesion disorder	(37)
c698C > T	4	p.T233M	Autosomal dominant	None reported	Age-related cerebellar atrophy causing ataxia in patients with SCA34	Erythrodermia variabilis, a skin lesion disorder	(38)
c.512T > C	4	p.I171T	Autosomal dominant	Retinitis pigmentosa	Age-related cerebellar atrophy causing ataxia in patients with SCA34	None reported	(39)
c.689delT	6	p.Ile230Metfs∗22, truncation	Homozygous recessive	Limited retinal examination but no functional retinal data reported	Seizures, intellectual disability, and early childhood mortality	Ichthyosis	(40)
c.646C > T	5	p.Arg216X, truncation	Homozygous recessive	Limited retinal examination but no functional retinal data reported	Seizures, intellectual disability, and early childhood mortality	Ichthyosis	(40)
c.78C > G	1	p.Tyr26∗, truncation	Homozygous recessive	Tortuos vessel in macular area with subtle macular changes	None reported	Ichthyosis	(41)

Although this review focuses on STGD3 and ELOVL4 mutation-associated disorders, recessive STGD1 (also known as fundus flavimaculatus) represents the more prevalent cases of juvenile-onset Stargardt macular dystrophy diseases (44, 45, 46, 47, 48, 49, 50, 51). Unlike STGD3 caused by autosomal dominant mutations in ELOVL4, STGD1 is caused by inheritance of recessive mutations in ATP-binding cassette, sub-family A, member 4 (ABCA4 or ABCR) (46, 52, 53). ABCA4 is exclusively expressed in retina photoreceptor cells and is essential for the visual cycle by catalyzing the translocation of specific phosphatidylethanolamines from the extracellular/lumenal to the cytoplasmic leaflet of membranes through the hydrolysis of ATP to generate retinoid substrates (54, 55). The retinoid substrates imported by ABCA4 from the extracellular or intradiscal (rod) membrane surfaces to the cytoplasmic membrane surface are reduced to vitamin A by *trans*-retinol dehydrogenase and then transferred to the RPE where it is converted to 11-*cis*-retinal in both rod and cone photoreceptor cells (56). Owing to its essential function in the visual cycle, mutations in ABCA4 are also associated with retinitis pigmentosa-19, cone-rod dystrophy type 3, early-onset severe retinal dystrophy, fundus flavimaculatus, and other macular degenerative diseases (46, 51, 57, 58, 59, 60, 61, 62, 63).

In this review, however, we will focus on *ELOVL4* mutations of the last decade and their related phenotypes and impacts on human health and disease in ELOVL4-expressing tissues.

## Different mutations in Elovl4 cause different tissue-specific disorders in humans

### Heterozygous ELOVL4 mutations affecting retinal structure and function

**Fig. 1:**
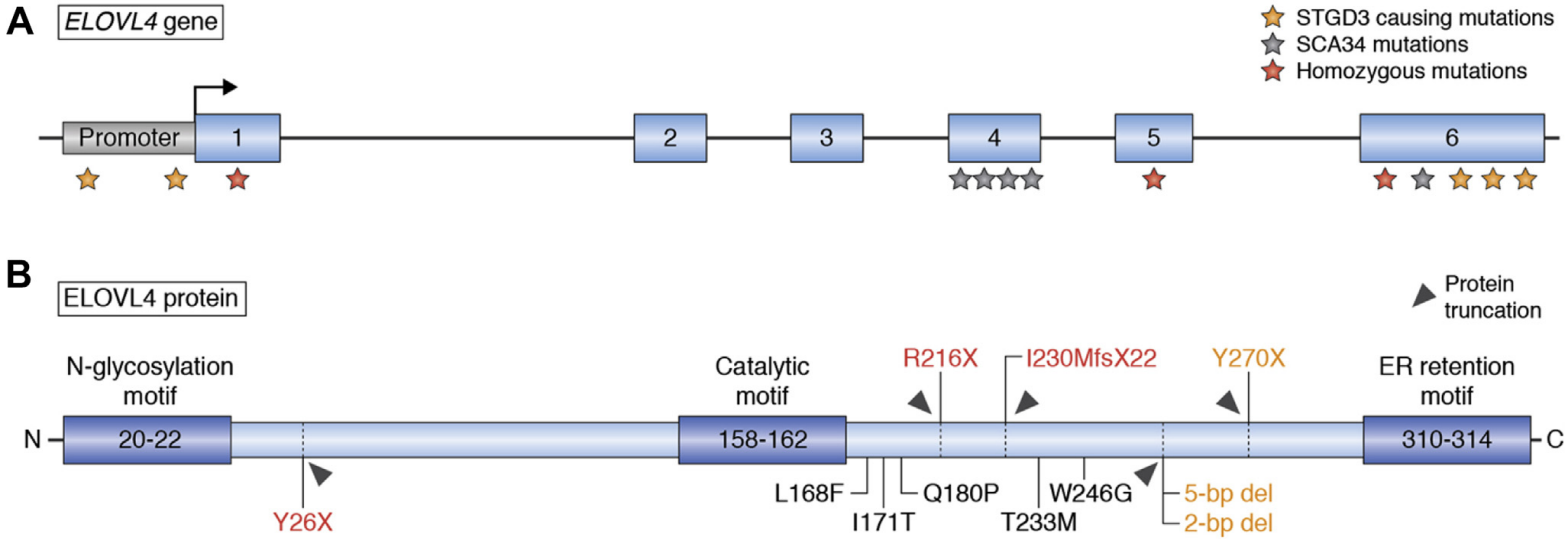
*ELOVL4* (A) and its translated protein (B) showing the location of the various mutations (A-B). All STGD3 causing mutations (orange) in exon 6 lead to a truncated protein, resulting in the loss of the ER retention motif. The two mutations in the promoter region of *ELOVL4* downregulate ELOVL4 expression based on luciferase activity and cause STGD3. Most of the SCA34 ELOVL4 (black) mutations cluster in the exon 4 region, except for an exon 6 mutation that produces a full-length protein with a single amino acid substitution. Homozygous mutations (red) produce an even more severe truncation of the protein, with an exon 1 mutation lacking the catalytic motif and ER retention motif. None of the mutations occur in the active region of ELOVL4, yet the STGD3 5 bp deletion mutant is enzymatically inactive due to loss of the ER targeting motif.

The initial ELOVL4 mutations (Fig. 1) that cause blindness in patients with STGD3 were reported in exon 6 of ELOVL4 (1, 2, 3, 4). Although these mutations lead to a truncated ELOVL4 protein that lacks VLC-FA biosynthesis, in the last decade Bardak *et al.* (64) reported two novel genetic base pair substitution mutations (c.814G > C and c.895A > G) in exon 6 as the cause of STGD3 (64). Donato *et al.* (34) then reported two point mutations (c. −236C > and c. −90G > C) (Fig. 1) in the promoter region of ELOVL4 in a patient with the retinal phenotypic characteristics of patients with STGD3. In 2020, a sporadic novel missense in ELOVL4, c.59A > G (p.N20S) variant, was reported in a Chinese patient with clinical manifestations similar to those of STGD1 (61). Of interest, apart from retinal degeneration, there are no reported brain and skin pathologies in patients with STGD3, as we previously reviewed (42).

The discovery by Donato *et al.* (34) that mutations in the ELOVL4 promoter cause STGD3 suggests that transcriptional regulation of ELOVL4 and its products may contribute to the quantity and quality of VLC-FA products synthesized by ELOVL4-expressing tissues, especially the retina, brain, and skin. Compared with the heterozygous mutations within the coding domain of ELOVL4 that cause a truncation or potentially altered protein structure and function, STGD3 pathology arising from single nucleotide polymorphisms (SNPs) in the promoter region of ELOVL4 further suggests that depletion of retinal VLC-FA due to mutant ELOVL4 activity could be the cause of retinal and other tissue-specific pathologies.

### Heterozygous ELOVL4 mutations affecting the brain cause age-related spinocerebellar ataxia with or without skin disorders

In the last decade, a number of different heterozygous mutations in different exons of the *ELOVL4* gene (Fig. 1) have been reported to cause age-related autosomal dominant spinocerebellar ataxia-34 (SCA34) with or without the skin condition erythrokeratodermia variabilis (35, 36, 37, 38, 39, 43). The first known heterozygous *ELOVL4* mutation to cause SCA34 and EKV was reported in a large French-Canadian family by Cadieux-Dion *et al.* in 2014 (35). Affected family members carry a transversion mutation, c.540G > C (p.L168F), in exon 4 of *ELOVL4*, which segregates with a skin phenotype consisting of early-onset patches of erythema and hyperkeratosis with the ataxia pathology manifesting in the fourth or fifth decade of life (35). Magnetic resonance imaging (MRI) of the brain of these patients revealed severe atrophy of the cerebellum and the pons (35). Some affected individuals showed cerebellar hypometabolism, as determined by brain fluorodeoxyglucose positron emission tomography (35).

The following year, another SCA34-causing mutation, c.736T > G (p.W246G) in exon 6 of *ELOVL4* (Fig. 1), was discovered in a Japanese family who also have selective degeneration of pontocerebellar tracts (hot cross bun sign) but did not have EKV like the French-Canadian patients (36). However, as in the French-Canadian family, the affected Japanese family members also showed age-related progressive ataxia, ocular movement disturbances, dysarthria, pyramidal tract signs, and pontocerebellar atrophy (36). The same year, Bourassa *et al.* (37) reported another heterozygous mutation, c.539A > C; p.G180P in exon 4 of *ELOVL4*, in a man in his thirties who developed progressive ataxia disorder starting in his mid-twenties and whose MRI showed cerebellar and pontine atrophy (37). Like the French-Canadian patients, this patient also had erythematous skin lesions on his forearms and legs during adolescence (37). These reports were followed by that of Bourque *et al.* of another novel heterozygous *ELOVL4* mutation, c. 698C > T, p T233M in exon 6 of ELOVL4, that caused both EKV and SCA34 in an English-Canadian woman with clinical features similar to the L168F mutation reported in the 32 affected members of the French-Canadian family (35, 38). The patient reported noticing ataxia during her teenage years but had early-onset skin pathologies starting around the age of four that progressed to localized skin thickening (38). Although oculomotor symptoms were reported in the patient, detailed ophthalmological fundus examination of the macula was not reported (38). In 2019, another heterozygous ELOVL4 mutation, c.698C > T (p.T233M), was discovered in a patient with multisystem neurodegeneration, including ataxia and EKV skin lesions (65). MRI of the brain of the patient and his father, who also presented with ataxia but no skin pathology, showed pontine and cerebellar atrophy as well as the hot cross bun sign as previously reported in patients with SCA34 with p. L198F and p. W246G ELOVL4 mutations (35, 36, 65). In 2020, Beaudin *et al.* (43) reported further characterization of the C.504G > C p.L168F mutation previously reported by Cadiuex *et al.* (35) in another French-Canadian family in which the mean age of ataxia onset was 47 years. Unlike the family reported by Cadiuex *et al.* (35), none of the nine clinically affected family members reported by Beaudin *et al.* (43) had EKV. Most of the affected members, however, exhibited horizontal nystagmus and saccadic pursuits, and one of the nine affected individuals had pisiform perimacular lesions (43). Further clinical characterization of the patients revealed cerebellar and pontine atrophy (four of six patients) and cruciform hypersignal in the pons (two of six patients). Fluorodeoxyglucose-positron emission tomography imaging revealed diffuse cerebellar hypometabolism in all five tested patients, with subtle parietal hypometabolism in three of them. The patients also exhibited cognitive deficits in executive functioning that were statistically significantly different from age- and education-matched controls (43).

As the list of heterozygous SCA34 ELOVL4 mutations grew (Table 1), in 2019 another ELOVL4 mutation, c.512T > C, p.I171T in exon 4 of *ELOVL4*, (Fig. 1) was reported in a family that presented with both SCA34 and retinal dystrophy characteristic of *ELOVL4* mutations that cause STGD3 (39). Ophthalmologic evaluation showed that four of the eight individuals in the family had retinal abnormalities consistent with retinitis pigmentosa (39). This was the first reported case where an individual with SCA34 pathology also presented with a retinal deficit from the *ELOVL4* mutation (39). It is also the first reported case where an *ELOVL4* mutation was linked to retinitis pigmentosa. Thus, even with the SCA34-causing ELOVL4 mutations, there are tissue-specific variabilities in the phenotypes reported. Of more importance, there are differences in the time of disease onset and rate of progression of the different tissue-specific disorders caused by different ELOVL4 mutations. Some patients with STGD3 seem to have early-onset vison loss (1, 2, 3, 4, 10, 34, 66), whereas patients with SCA34 with or without EKV develop relatively slow progression of ataxia, with average onset in adulthood (35, 36, 37, 38, 39, 43, 65).

### Homozygous ELOVL4 mutations cause severe intellectual disability, seizures, and early childhood death

To date, three different homozygous mutations in ELOVL4 have been reported to cause very severe pathologies that affect the skin and brain, resulting in early childhood mortality (40, 41). Overall, inheritance of the homozygous ELOVL4 mutations, c.78C > G; p.Tyr26∗, and c.646C > T, p.Arg216X (41) in exon 5, and c.690del p.Ile230Metfs∗22 in exon 6 of ELOVL4 (40), causes dry, scaly, and thickened skin disorders (ichthyosis), intellectual disability, seizures, hypertonia, and premature death (40, 41). An important characteristic of the neuroichthyotic disorders in these patients is that it shares clinical features resembling Sjogren-Larsson syndrome (MIM 270200) (40). These findings suggest that there are potentially other novel ELOVL4 mutations that have not yet been reported because the patients may have been assigned to other clinical categories based on their phenotype. We therefore speculate that new ELOVL4 mutation variants will be discovered and reported.

Consistent with the human pathologies, mice that are homozygous for the STDG3 alleles or homozygous knockout of mouse *Elovl4* die at birth (23, 31, 32, 33). Transgenic expression of wild-type ELOVL4 in the skin using skin-specific promoters rescued these mice from neonatal lethality (67, 68). However, the skin-rescued mice develop seizure-like phenotypes similar to the symptoms seen in humans homozygous for ELOVL4 and die by postnatal day 21 owing to the lack of brain ELOVL4 VLC-FA products (68), which supports the essential role of ELOVL4-synthesized lipids in health and disease. How the different mutations in *ELOVL4* cause such different tissue-specific phenotypes and rates of progression of disease is a puzzling question that remains to be answered.

## Deciphering the mutant ELOVL4 disease pathology by understanding the structure and tissue distribution of ELOVL4

**Fig. 2:**
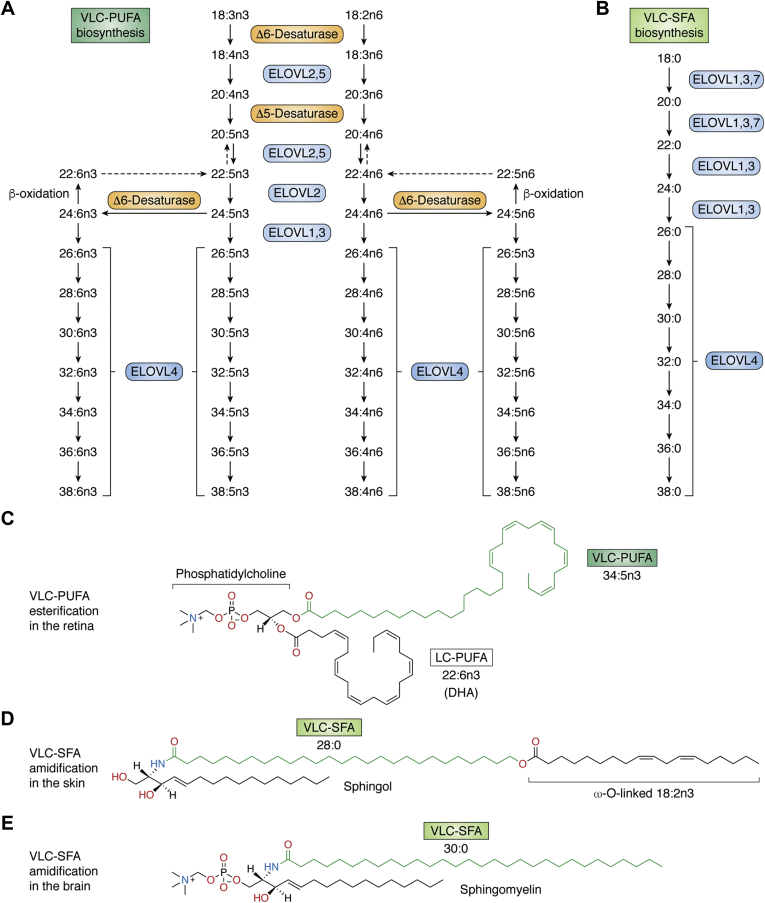
Biosynthesis of VLC-PUFA and VLC-SFA. A: Schematic in vivo biosynthetic pathway from 18:3n3 and 18:2n6 mediated by ELOVL4 and other ELOVL family proteins. Desaturase and elongation steps are consecutively performed by fatty acid desaturase-1 (FADS1 or Δ5 desaturase), fatty acid desaturase-2 (FADS2 or Δ6 desaturase), and ELOVL1-5. Although some elongases are specific for a single step, others are nonspecific or multifunctional and act at several steps (e.g., human ELOVL5 and murine ELOVL2). Panel A is an adapted reproduction from Man Yu *et al.* (69). ©2012 by the American Society for Biochemistry and Molecular Biology, Inc. B: VLC-SFA biosynthesis pathway. Elongation steps from 18:0 to VLC-SFA by the different ELOVLs. C: Example of VLC-PUFA esterification in the retina: phosphatidylcholine containing the VLC-PUFA, 34:5n3 and the LC-PUFA, 22:6n3 (DHA). D: Example of VLC-SFA amidification in the skin: ω-O-acylceramide containing the VLC-SFA, 28:0 ω-O-linked with 18:2n6. E: Example of VLC-SFA amidification in the brain: sphingomyelin containing the VLC-SFA, 30:0. Panels C–E, adapted from Hopiavuori *et al.* (70) used with permission.

There are seven members of the fatty acid elongase (ELOVL1-7) family that work in collaboration with other fatty acid biosynthetic enzymes to elongate specific chain lengths of fatty acids in a tissue-specific manner (Fig. 2) (70, 71, 72, 73, 74, 75, 76). These enzymes are transmembrane resident proteins of the endoplasmic reticulum (ER) within which they elongate both saturated and unsaturated fatty acids with specificity toward a particular fatty acid chain length (77, 78). ELOVL4 specifically synthesizes both VLC-SFA and VLC-PUFA from C26 fatty acids (Fig. 2A, B) (28, 69). These enzymes contain three characteristic motifs, namely, (1) an N-glycosylation consensus motif at the N terminus, (2) a catalytic histidine core (HXXHH), and (3) an ER retention/retrieval motif (KXKXX) located at the C terminus. Site-specific mutations within these motifs in cell culture experiments established that the activity of ELOVL enzymes is dependent on an intact catalytic histidine core and the ER retention/retrieval motif (29, 30, 79). Despite the ability of ELOVL4 to elongate both PUFA and SFA, the quantitative and qualitative distribution of VLC-PUFA and VLC-SFA varies in the different ELOVL4-expressing tissues. In terms of its tissue distribution, our current knowledge is that ELOVL4 is expressed in the retina, Meibomian glands, brain, skin, and testes (1, 28, 80, 81). However, there is the probability that, as we continue to study the ELOVL4 protein, with time, we may discover other tissues that express it under specific conditions. In the eye, the highest ELOVL4 expression is seen in the rod and cone photoreceptor inner segments and the outer nuclear layer, where VLC-PUFA are the main ELOVL4 products that are selectively incorporated into the sn-1 position in phosphatidylcholine (PC) with DHA esterified at the sn-2 position (21, 28, 80, 82, 83, 84, 85, 86). Although VLC-PUFA are the main products of ELOVL4 in the retina and testes in which they are incorporated into PC and sphingolipids, respectively, VLC-SFA are predominantly synthesized in the skin, brain, and Meibomian glands and incorporated into sphingolipids (Fig. 2D, E). Even within the retina, VLC-PUFA are incorporated mainly into PC lipid species (Fig. 2C), whereas in the testes and sperm, VLC-PUFA are incorporated into sphingolipids suggesting that different tissues have different specific needs for ELOVL4 products. Also, in the eye, ELOVL4 is expressed within the Meibomian glands, which produce a lipid mixture of meibum that mixes with aqueous tears produced by the lachrymal glands to form the tear film that covers the cornea (81). This lipid mixture contains VLC-SFA produced by ELOVL4 that are incorporated into (O-acyl)-ω-hydroxy FA (87, 88, 89, 90, 91, 92). Reduced VLC-SFA relative to other lipids may alter the rate of evaporation of the tear film. Of significance, changes in the quality and quantity of the lipids secreted by the Meibomian glands are believed to be an underlying cause of the evaporative form of dry eye disease (81, 93). Meibum is believed to be excreted spontaneously or when blinking. It is interesting that STGD3 5 bp deletion heterozygous mice, likely having reduced VLC-SFA in their tear film, exhibited increased blinking of the eyelids and an aversion to keeping their eyes fully open and showed an evaporative type of dry eye disease phenotype (81). This supports the important role of ELOVL4-synthesized VLC-SFA in ocular health and the prevention of rapid evaporation of the eye's tear film, which needs further studies to unravel the role of ELOVL4 in age-related dry eye diseases.

In the brain, the ELOVL4 products are essential for normal brain function such that depletion of brain ELOVL4 leads to seizures and premature death in rodents and humans (40, 41, 68). We have shown that ELOVL4 is expressed in neurons in the brain, with the most abundant expression in most neurons by embryonic day 18 in mice (94). We detected 28:0 and 30:0 VLC-SFA, which are incorporated into complex sphingolipids and enriched in synaptic vesicle preparations from baboon brains (68). We showed increased synaptic release kinetics in the hippocampal neurons from homozygous *Elovl4*^*Stgd3/Stgd3*^ mice compared with neurons from wild-type littermates (68). Supplementing the primary neurons from the *Elovl4* homozygous mice with 28:0 and 30:0 rescued the synaptic release rates to wild-type levels (68). Thus, brain VLC-SFA seem to modulate presynaptic transmitter release kinetics that requires further scientific investigation.

The expression of ELOVL4 in the skin is important for biosynthesis of VLC-SFA that are essential for skin barrier function and overall survival (31, 95). The essential role of VLC-SFA for skin function and survival is unequivocally demonstrated in homozygous STGD3 knockin mice and homozygous global *Elovl4* knockout mice that die at birth (23, 31, 32, 33) and in humans with homozygous ELOVL4 mutations who die within the first decade of life from a number of neurological and skin disorders (40, 41). We and others have established the essential role of ELOVL4 and its VLC-FA products for life by demonstrating that the expression of ELOVL4 in the skin rescues the skin defects that cause neonatal lethality in homozygous STGD3 knockin mice (67, 68).

## What factors determine the different tissue-specific disorders caused by different ELOVL4 mutations?

The tissue-specific biosynthesis of VLC-PUFA relative to VLC-SFA in different ELOVL4-expressing tissues is likely determined by tissue-specific factors, such as availability of substrates and/or proteins that interact with ELOVL4. In the retina, the biosynthesis of VLC-PUFA is likely due to the higher presence of PUFA substrates, whereas in the skin and Meibomian glands, the preference toward VLC-SFA biosynthesis is probably due to the lack of PUFA precursors and presence of VLC-SFA precursors (Fig. 2A, B). DHA and arachidonic acid (20:4n6) (AA) are the predominant PUFA in the retina and brain (96, 97, 98, 99, 100, 101, 102, 103, 104, 105). However, to date, despite our exhaustive search, we have not been able to detect VLC-PUFA in the normal brain, but we have readily detected VLC-PUFA in retinal PC and sperm sphingolipids and VLC-SFA in skin (28, 82, 83, 84, 106, 107, 108, 109). This suggests VLC-PUFA and VLC-SFA may be differentially metabolized in the brain and in the retina. Since fatty acid oxidation occurs in peroxisomes when fatty acid chains are too long to be oxidized by the mitochondria, the presence of VLC-PUFA in the brain and other tissues of Zellweger spectrum disorders, but not in normal brain, is attributed to impaired peroxisome dysfunction (110, 111, 112). On the other hand, it may be argued that the presence of VLC-PUFA in the brains of Zellweger spectrum disorders suggests that, in the normal brain, VLC-PUFA are efficiently utilized for brain function, hence their lack of accumulation. We need genetic animal models and *in* *vitro* studies to test these proposals.

Studies have shown that some fatty acids may be utilized more for energy purposes. DHA is demonstrated to be a relatively poor substrate for beta-oxidation in the mitochondria and peroxisomes (113, 114, 115). EPA is present in the retina and brain at low levels probably because they are more readily metabolized or oxidized than DHA; as a result, there is limited retroconversion of DHA back to EPA in peroxisomes (113, 114, 116). To sustain the high energy needs of the brain, substantial EPA may therefore not be sufficient for significant FA elongation to VLC-PUFA, since FA elongation and FA catabolism cannot occur simultaneously (115, 117). Coincidentally, based on our *in* *vitro* studies, we showed that EPA is a preferred substrate for VLC-PUFA biosynthesis by ELOVL4-expressing cells treated with equimolar concentrations of DHA and EPA or DHA and AA (69). These findings are consistent with the discovery that in Atlantic salmon (*Salmo salar*), *Elovl4* synthesized greater amounts of VLC-PUFA from EPA than from AA and DHA (118). Also, in the retina, DHA molecules are principal components of retinal PC-containing VLC-PUFA and hence may not undergo extensive metabolism while incorporated into phospholipids (119). For instance, when vitreal fluid of rat eyes was injected with tritium-labeled [^3^H]-DHA, 90% of the [^3^H]-DHA remained as 22:6n-3 in the phospholipids after 48 h *in vivo* metabolism (120). These observations indicate that DHA is likely utilized more for structural purposes, influencing fluidity of the membrane without further metabolism (121). Therefore, despite the presence of relatively high DHA in the brain, it may be serving more as structural and signaling elements and increasingly not available as substrates for ELOVL4 elongation into VLC-PUFA (122). As we previously proposed (68), absence of VLC-PUFA in the normal brain could be due to the fact that any VLC-PUFA produced are efficiently biotransformed into Elovanoids that serve a neuroprotective signaling function (123). This thought is further supported by the fact that the absence of Adiponectin receptor 1, a receptor necessary for DHA uptake into the retina, leads to depletion of not only DHA but also Elovanoids and their VLC-PUFA precursors 32:6n3 or 34:6n3 (124). Also, sufficient availability of a particular fatty acid may interfere with the elongation of another type of fatty acid in a given tissue. For example, using liver microsomal assays, it was reported that the chain elongation of linoleic acid (18:2n6) was more than 60% inhibited by saturated fatty acids myristic acid (14:0) and pentadecanoic acid (15:0) (125). This form of competitive inhibition may play a role in the abundance of VLC-SFA or VLC-PUFA in a tissue-specific manner.

Another factor that could determine the predominant product of ELOVL4 in a tissue-specific manner may be the ELOVL4 enzyme elongation complex substrate discrimination as determined by specific interacting proteins within a given tissue. Saturated and polyunsaturated FA have different physiochemical properties, with straight-chained saturated fatty acids being able to pack closely, whereas PUFA, owing to the presence of double bonds, occupy more space. The double bonds of PUFA cause them to have bent conformations along the acyl chain, so they do not pack tightly. ELOVL1-7 exhibits characteristic substrate specificities toward acyl-CoAs. Some elongases, such as ELOVL6 and ELOVL7, have preferential elongation activity toward saturated fatty acids rather than PUFA. Thus, it is possible that differential expression of the type of ELOVL proteins in a given tissue could determine the type of fatty acid synthesized within that tissue (72). *In vitro* studies showed that elongation by ELOVL6 was limited to long-chain saturated and monounsaturated fatty acyl-CoAs with chain lengths of 12–16 carbons. This finding shows that an increase in carbon length and degree of unsaturation in the FA limit the ability of ELOVL6 to elongate it (126). Although VLC-SFA biosynthesis requires expression of combinations of ELOVL1, 3, 4, 6, and 7 in cells, ELOVL2 and ELOVL5 have substrate preference for PUFA by elongating C18–C20 PUFA (127, 128). However, only ELOVL2 further converts 22 carbon PUFA n3/n6 to 24 carbon PUFA n3/n6 (127). Subsequently, the 24 carbon PUFA can be further elongated into 26 carbon PUFA (which are substrates of ELOVL4) by ELOVL1 and 3. *In* *vitro*, both ELOVL2 and 5 enzymes elongated EPA (20:5n3, a substrate common to both ELOVL2 and 5) to DPA (22:5n3). However, only ELOVL2 further elongated the DPA product to 24:5n3 (127).

Using a yeast expression system, the cysteine at position 217 in ELOVL2 and a tryptophan at the corresponding position in ELOVL5 have been identified as the molecular reason for the differences in activity toward elongation of DPA to 24:5n3. As a result, C217W-ELOVL2 mutant loses the ability to convert DPA to 24:5n3 while retaining enzymatic activity to elongate EPA to DPA (127).

**Fig. 3:**
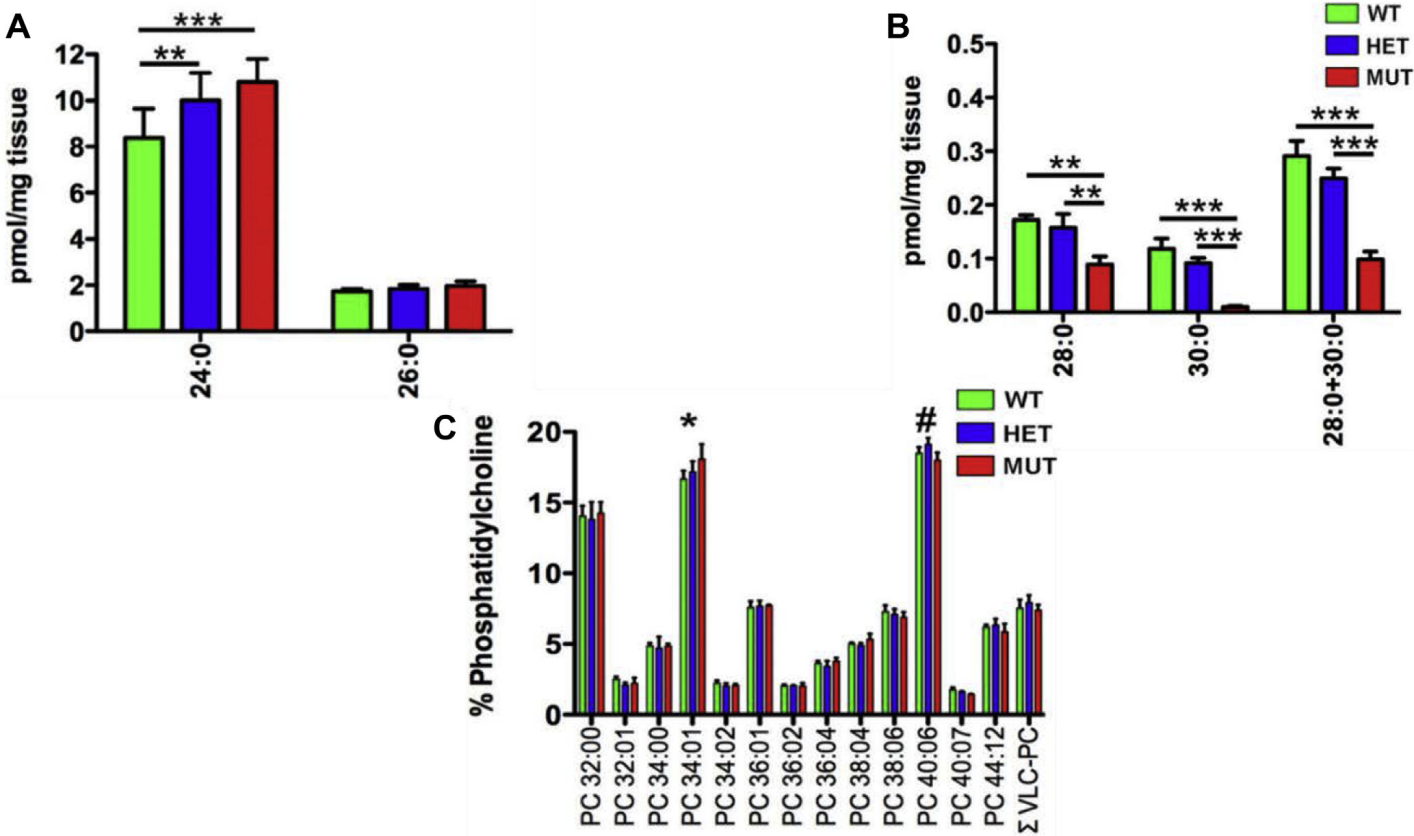
The W246G mutation in ELOVL4 impairs VLC-SFA synthesis but retains the ability to synthesize VLC-PUFA. A: Analysis of VLC-SFA in skin. Levels of VLC-SFA (28:0 and 30:0) and total VLC-SFA (28:0 + 30:0) were significantly reduced in the skin of MUT rats compared with WT and HET rats. B: Levels of 26:0, the direct precursor for VLC-SFA synthesis, did not differ significantly across genotypes. However, levels of 24:0 were significantly elevated in the skin of HET and MUT rats compared with WT rats. (Data shown as mean ± SD. Analysis by one-way ANOVA with Tukey's post hoc test. ∗*P* < 0.05; ∗∗*P* < 0.01; ∗∗∗*P* < 0.001). C: VLC-PUFA were detected specifically in the phosphatidylcholine fraction (PC), but total VLC-PUFA levels showed no differences among WT, HET, and MUT rat retinas. However, significant differences were detected in non-VLC-FA species (PC 34:01 and PC 40:06) among genotypes.

We recently generated a knock-in Long Evans rat model of SCA34 (SCA34-KI) that expresses the 736T > G (p.W246G) form of *ELOVL4* that causes human SCA34. Analyses of retina and skin lipids of the SCA34-KI rats showed that the W246G ELOVL4 mutation selectively impaired synthesis of VLC-SFA in the skin, but not retinal VLC-PUFA (Fig. 3) (129). Consistent with the human SCA34 pathology, the homozygous SCA34 rats that developed progressive motor learning defects with age were significantly smaller than their heterozygous and wild-type control littermates and developed reddish pink scaly skin similar to the human EKV pathology (129). Analyses of VLC-SFA in skin showed significantly reduced levels of 28:0 and 30:0 in homozygous mutant rats (MUT) compared with wild-type (WT) and heterozygous (HET) ones (Fig. 3A, B). We also showed the levels of 24:0 were significantly elevated in the skin of HET and MUT rats compared with WT rats (Fig. 3A). Of interest, total retinal VLC-PUFA-PC levels showed no differences among the WT, HET, and MUT rat retinas (Fig. 3C), which suggests that the W246G mutation affected skin VLC-SFA biosynthesis but had no effect on retinal VLC-PUFA biosynthesis. Recent studies by Parisi *et al.* (130, 131) demonstrated that increased levels of C24 fatty acids can disturb membrane integrity more readily than C16 fatty acids. Using molecular dynamics simulations, they showed that 24:0 can interdigitate between leaflets of the lipid bilayer, whereas C16 FA does not, and that increases in the levels of saturated VLC-FA result in cellular membrane permeabilization during necroptosis (130, 131). They proposed that dysregulation of VLC-SFA can cause membrane disruption either by directly disrupting membrane packing or facilitating permeabilization by targeting proteins to the plasma membrane. This observation could explain the EKV-like skin disorders seen in humans and in our SCA34 rat model (129, 130, 131). Taken together, these results suggest that the W246G ELOVL4 protein may have substrate preference for PUFA but not SFA, which would support the hypothesis that ELOVL4 mutations that affect the skin and brain but not the retina impair the synthesis of VLC-SFA, which are the ELOVL4 products of the skin, and not VLC-PUFA, the ELOVL4 products in the retina. It is also possible that mutations in ELOVL4 that significantly change the conformation of the protein structure and its active site may steer ELOVL4 substrate preference for either SFA or PUFA, hence the tissue-specific disorders caused by the different ELOVL4 mutations.

## Roles of VLC-FA-containing lipids in retinal function

### Genetic and aging components that could affect retinal fatty acid biosynthesis and cause retinal pathologies

A pathologic hallmark of STGD3 is the presence of lipid-containing residue lipofuscin in the RPE, RPE atrophy, and macular degeneration (10, 11, 66). VLC-PUFA are the main products of ELOVL4 in the retina and testes, whereas VLC-SFA are predominantly synthesized in the skin, brain, and Meibomian glands. Although the role of VLC-PUFA in the retina is not completely understood, the current data suggest that retinal VLC-PUFA confer fluidity on retinal outer segment membranes and are essential for synaptic function and overall health and function of the retina and RPE (132, 133, 134). Indeed, Liu *et al.* (135) reported the presence of 21 different VLC-PUFA belonging to six different groups in human retina and RPE/choroid. These groups include n-3 and n-6 VLC-PUFA from C24 to C34 with variable double bonds (135). They showed that, in the whole retina, the levels of 28:4n-6, 30:6n-3, 30:5n-3, 32:4n-6, and 32:5n-3 peaked in middle age, with the levels of 24:6n-3, 26:4n-6, 28:4n-6, 28:5n-3, 30:4n-6, 32:5n-6, 32:6n-3, 32:5n-3, and 34:4n-6 being significantly higher in normal old age retinas compared with the age-matched AMD group (135). Compared with the retina, one-tenth of C30–C34 VLC-PUFA were detected in RPE/choroid, which also showed a trend in age-related changes in VLC-PUFA, with levels peaking in middle age donor eyes and severely decreasing in AMD donors (135). These findings suggest that, even in the normal eye, normal aging affects the levels of retinal VLC-PUFA.

An important finding from the Liu *et al.* study (135) is the decrease in C24–C26 PUFA in AMD retinas compared with age-matched normal retinas. This decrease was proposed to be likely due to lower levels of precursors such as 22:4n-6 and 22:5n-3 in AMD retinas or impaired enzymatic processing (135). In a follow-up study (136) to the Liu *et al.* study from 2010, the Bernstein lab collected and analyzed donor eyes and serum from patients with AMD (n = 15; aged 73–91 years) and non-AMD control patients (n = 21; aged 74–88 years). They showed that dietary intake of LC-PUFA, which are precursors for VLC-PUFA, influenced the retinal lipid profile and confirmed that AMD eyes have decreased VLC-PUFA by showing that retinal VLC-PUFA levels were significantly lower in AMD eyes compared with levels in age-matched control eyes (136). They proposed that serum LC-PUFA levels are good predictors of retinal LC-PUFA and VLC-PUFA levels (136). Furthermore, they explored the potential contribution of TT and CT variants of *ELOVL4* (*rs3812153*) to AMD pathogenesis. They reported that AMD donors with the CT *ELOVL4* variant allele had one form or the other of pigmentary irregularities, soft drusen deposits (>63 and ≤125 μm), geographic atrophy, and choroidal neovascular membranes compared with carriers with the lower-risk TT allele variant (136). However, consistent with previous studies, they did not find a statistically significant correlation between the lower-risk TT allele compared with the CT *ELOVL4* variant and macular degeneration (136, 137, 138). In light of the new discoveries of different ELOVL4 mutations causing different tissue-specific disorders in humans, researchers should consider including analyses of the CT and TT variants in *ELOVL4* in patients with AMD in future studies. In line with the essential role of VLC-PUFA in retinal function, the report by Donato *et al.* (34) that SNP in the human ELOVL4 promoter causes STGD3 suggests that loss of ELOVL4 enzymatic activity, and therefore a decrease in VLC-FA products, could cause an age-related decline in visual function, RPE atrophy, and photoreceptor death.

Indeed, it is now emerging that age-related hypermethylation of genes involved in retinal fatty acid metabolism that suppresses transcription of these genes potentially contributes to some of the age-related decrease in retinal LC-PUFA and VLC-PUFA (139). ELOVL2 is expressed in the retina and plays critical roles in the biosynthesis of LC-PUFA that are essential precursors for VLC-PUFA biosynthesis (140). *ELOVL2* has emerged as a prominent aging biomarker (139, 141, 142). Age-related increased methylation in *ELOVL2* occurs not only in humans but also in mouse retina and liver, where it is highly expressed (140). Recent studies have also reported that genetic variations in ELOVL2 affect the level of blood EPA and DHA, thereby providing an inconsistent degree of protection from cardiovascular disease (143). Carriers of the SNPs rs3734398, rs2236212, and rs953413 in *ELOVL2* have lower proportions of plasma DHA than do noncarriers at baseline, which could be improved by dietary EPA and DHA supplementation (143). These findings suggest that, in the general population, age-related methylation of the regulatory regions of *ELOVL2* coupled with SNPs in *ELOVL2*, and potentially in other ELOVLs and fatty acid desaturases that are essential for fatty acid metabolism, could be one of the possible molecular explanations for the age-related decrease in retinal LC-PUFA and VLC-PUFA levels.

Recently, the Skowronska-Krawczyk lab showed that the *Elovl2* promoter region is increasingly methylated with age in the retina and caused an age-dependent decrease in ELOVL2 expression in the liver and retina (140). Their analyses of the fundus of wild-type C57BL/6J mice showed age-dependent accumulation of autofluorescent aggregates and a decrease in visual function at 24 months, which could be reversed *in* *vivo* by intravitreal injection of 5-Aza-2′-deoxycytidine (140). To further understand how downregulation of ELOVL2 could cause age-related changes in visual function in the mouse retina, they used the CRISPR-Cas9 technology to generate mutant *Elovl2* knockin mice that encode a cysteine-to-tryptophan substitution (C234W) in ELOVL2 (140). The C234W ELOVL2 mutation caused selective inhibition of elongation of C22-PUFA to C24-PUFA, while its activity toward other ELOVL2 substrates was retained (140). The heterozygous *Elovl2*^*C234W*^ mice were fertile and bred to produce homozygous *Elovl2*^*C234W*^ mice that developed normally. However, fatty acid analyses of the retina and liver of the *Elovl2*^*C234W*^ mice revealed accumulation of 22:5 fatty acids and decreased levels of 24:5 and 22:6 fatty acids, which supports loss of ELOVL2 enzymatic activity toward C22 elongation (140). What is remarkable about the loss of ELOVL2 activity and decrease in the C24 fatty acids in the retina of these mice is that the homozygous *Elovl2*^*C234W*^ mice exhibited an accelerated aging phenotype as determined by a decline in retinal electrophysiological responses consistent with premature visual dysfunction and showed well-established aging markers related to autofluorescent deposits by 6 months of age (140). The results of age-related pathological changes in the wild-type C57BL/6J mouse retina induced by age-related hypermethylation of the *Elovl2* promoter were similar to the changes observed in the *Elovl2*^*C234W*^ mouse model (140). These results suggest that age-related dysregulation of retinal fatty acid biosynthetic genes contributes to some forms of age-related retinal degenerative diseases. One factor that was not studied in the *Elovl2*^*C234W*^ mouse was the potential contribution of the mutant C234W ELOVL2 protein to the pathology observed. We expect that, in future experiments, if the homozygous *Elovl2*^*C234W*^ mice are fed the missing fatty acids and are shown to be rescued from the accelerated aging phenotype due to increased retinal LC-PUFA and VLC-PUFA levels, this will validate the essential role of LC-PUFA and VLC-PUFA in retinal and brain function.

It must, however, be emphasized that the essential role of LC-PUFA and especially VLC-PUFA in the retina is still not completely understood. It is known that visual function and the accumulation of autofluorescent aggregates in the retina are highly correlated with aging (144, 145, 146, 147, 148, 149). In heterozygous *Elovl4* knockout (*Elovl4*^*+/−*^) animal models that lack expression of the STGD3 mutant ELOVL4 (25, 150) protein, there are no abnormal changes in rod and cone morphology or decline in photoreceptor function compared with transgenic mouse models expressing the STGD3 ELOVL4 protein (19, 22, 26). For instance, retinas from *Elovl4*^*+/−*^ were reported by two independent groups to have normal retinal morphology up to 22 months (25, 150). Furthermore, immunohistochemistry and confocal microscopy of cone and rod conditional knockout mouse retinas revealed normal retinal outer segment morphology until 10 and 15 months, when outer nuclear layer thickness measurements showed a mild photoreceptor cell loss in comparison with normal controls (134). Also, using a *Chx10-Cre* to conditionally delete *Elovl4* from photoreceptor cells, Bennett *et al.* (132, 133) showed normal retinal morphology in cKO mice and WT controls up until 12 months of age, when there was a small but significant loss of rod photoreceptor nuclei and synaptic vesicle changes in the cKO mice compared with WT controls. However, following the huge decline in VLC-PUFA there was a significant reduction in rod responses compared with wild type as assessed by electroretinography (ERG). These results on the surface would suggest that VLC-PUFA are not critical for photoreceptor structure. However, as reported by the Bernstein lab (135, 136) and in our own studies in rodents (151, 152), the levels of VLC-PUFA in the rod-dominant rodent retina are much higher than in cone-dominant retinas (151) and in the human macula (153). It is possible that differences in the amount of VLC-PUFA in the rodent retina relative to the human retina could account for the reason why mouse *Elovl4* models without mutant ELOVL4 expression do not show significant defects in retinal function and structure. The finding that loss of ELOVL4 products results in rapid decline in visual function, RPE atrophy, and photoreceptor death in STGD3 animal models strengthens the relationship between VLC-PUFA and macular degeneration (132, 133).

Another important reason for the need to understand the contribution of VLC-PUFA to retinal function is that, despite considerable epidemiological and animal studies implicating the essential role of LC-PUFA in retinal function, the negative results of the Age-Related Disease Study 2 (AREDS2) that suggest that DHA supplementation is not able to attenuate progression of macular degeneration were unexpected (154, 155). We have shown that, compared with well-characterized rod-dominant retinas, cone-dominant retinas have significantly less DHA and VLC-PUFA in whole retinas and outer segment (OS) membranes, which is consistent with previous studies that showed less DHA in human macula relative to the peripheral retina (151). Cone photoreceptors intrinsically have low levels of LC-PUFA and VLC-PUFA, which suggests that genetic and age-related epigenetic modifications that affect genes involved in LC-PUFA and VLC-PUFA biosynthesis to further decrease the levels of these fatty acids could increase the susceptibility of the macula to age-related RPE and photoreceptor cell degeneration (151). Also, the evidence of age-related epigenetic regulation of ELOVL2 (140) and its effect on lipid biosynthesis combined with Donato *et al.*'s (34) report of SNPs in the ELOVL4 promoter causing STGD3 suggest that age-related changes and genetic factors that affect the expression of lipid biosynthetic genes play a role in retinal health. Thus, the role of age-related depletion/loss of VLC-PUFA in the contribution to STGD3 and AMD pathologies cannot be ruled out and merits further investigation.

### Biochemical protective roles of VLC-PUFA-derived bioactive lipids in the retina

Oxidative stress is a major risk factor for AMD (156). The retina is constantly exposed to oxidative stress, and any form of uncompensated oxidative stress can trigger pathways that can lead to photoreceptor cell death (156, 157, 158). Recent breakthrough studies based on our discovery that ELOVL4 is essential for the biosynthesis of VLC-PUFA have shown that VLC-PUFA-derived bioactive lipids may protect photoreceptors against oxidative stress (123, 159). Studies by the Bazan lab showed that oxygenated derivatives of VLC-PUFA called “*Elovanoids*” serve as a survival signal during uncompensated oxidative stress (159). The Elovanoids ELV-N32 and ELV-N34, which are dihydroxylated derivatives of 32:6n-3 and 34:6n-3, respectively, were discovered to protect RPE cells from hydrogen peroxide-induced cell death by upregulating the expression of prosurvival proteins like Bcl-2 and Bcl-xL and downregulating proapoptotic proteins Bax, Bim, and Bid (159). This finding shows that the reduction in the levels of VLC-PUFA-derived prosurvival mediators may play a role in photoreceptor death in many retinal degenerative diseases in which the retinal lipidome is affected. For instance, knockout of Adiponectin receptor 1, a key regulator of DHA uptake and retention in the retina, in mice leads to depletion of DHA (C22:6n3), 32:6n3 or 34:6n3, with ELV-N32 and ELV-N34 being undetectable even when subjected to uncompensated oxidative stress conditions.

Also, in recent studies, the sodium-dependent lipid transporter *Major Facilitator Superfamily Domain Containing 2-a* (*Mfsd2a*) has been demonstrated to be a major transporter of DHA across the blood-retina barrier into the retina, as well as DHA transport into the brain (160, 161, 162, 163). Homozygous knockout of *Mfsd2a* (*Mfsd2a*^*−/−*^) mice had about half the level of normal DHA and other fatty acids containing 22 and more carbons in the retina. There was also a corresponding decrease of about 57% VLC-PUFA in the retinas of these mice, which implicates the deficit of ELV-N32 and ELV-N34, despite normal *Elovl2* and *Elovl4* mRNA expression levels (159, 160). The effect of the decline of these lipids resulted in about 30% photoreceptor cell death by 6 months of age compared with WT littermates of the same age (160). Surviving photoreceptors retained normal ultrastructure, and single-cell recordings from the surviving rods of *Mfsd2a*^*−/−*^ mice showed indistinguishable amplitudes and kinetics of the light responses compared with wild-type littermates (160). However, Wong *et al.* (162) reported a reduction in outer rod segment length, disorganized outer rod segment discs, and mislocalization of and reduction in rhodopsin early in postnatal development, without loss of photoreceptors, in the *Mfsd2a*^*−/−*^ mice. Although these *Mfsd2a*^*−/−*^ mice had normal visual function as evaluated by ERG, their developing eyes were reported to have activated microglia and upregulation of lipogenic and cholesterogenic genes, which were attributed to adaptations to loss of DHA lysophosphatidylcholine transport (162). These findings support the essential role of DHA in photoreceptor development and function (162).

Taken together, these results suggest that the reduction in retinal LC-PUFA and VLC-PUFA plays a major role in retinal disease pathogenesis (140, 162). Results from these studies suggest that it is possible that decreased LC-PUFA and VLC-PUFA, the levels of which are regulated by fatty acid elongase and desaturase enzymes, contributes to a nonexudative form of AMD through gradual RPE atrophy and photoreceptor cell death. The implication here is that functional lipid biosynthetic genes are essential for biosynthesis of lipids that play protective roles in the retina and other neuronal tissues. If so, combined dietary supplementation of EPA and DHA, which are critical to maintain retinal structure and function, with VLC-PUFA may be beneficial in attenuating rescue of retinal functional deficits in AMD.

## Role of ELOVL4 mutant proteins in diseases of the retina *in vivo*: effect of STGD3 protein on photoreceptor health

Photoreceptor and RPE dysfunction is a common feature in STGD3 disease in humans (1, 10, 11, 66). A number of *in* *vivo* studies using different STGD3 and *Elovl4* knockout mouse models confirmed mislocalization of the STGD3 protein and loss of its enzymatic activity (19, 20, 22, 23, 24, 26, 164, 165). The first transgenic STGD3 mouse models reported expression of increasing copy numbers of the human STGD3 mutant ELOVL4 protein in both rods and cones using interphotoreceptor retinoid-binding protein promoter in wild-type mice that have two endogenous mouse *Elovl4* wild-type alleles (22). In these mouse models, the rate of retinal degeneration corresponds with the copy number of the human STGD3 mutant protein expressed (22). Our lipidomic analyses of the retinas of some of these transgenic mice at a stage where there was no significant retinal degeneration did not show significant loss of retinal VLC-PUFA (166). This suggests that biosynthesis of retinal VLC-PUFA by endogenous wild-type mouse ELOVL4 is not affected by the transgene expression in younger animals (166). This finding further suggests that retinal degeneration in the TG3 > TG2 > T1 mouse could be due to the overexpression of mutant human ELOVL4 causing retinal and RPE pathologies (22). Furthermore, in the heterozygous STGD3 5 bp deletion knockin mouse model (*Elovl4*^*WT/Mut*^), Vasireddy *et al.* (26) reported a significant decrease in the number of cones expressing S-opsin starting at 6 months compared with control animals. At 18 months, the number of cones expressing S-opsin continued to decrease, whereas there was no difference in the number of cones expressing M-opsin. McMahon *et al.* (23) also generated a heterozygous STGD3 knockin mouse model (*Elovl4*^*Wt/Stgd*3^). They reported no evidence of morphological changes or significant reduction in the length of photoreceptor outer segments or the number of photoreceptor cells of *Elovl4*^*Wt/Stgd3*^ mice compared with WT controls (23). The main difference between the Vasireddy and McMahon studies was in the age of the animals analyzed and the approach used in generating the knockin animals (23, 26). Work done in all other transgenic models expressing the mutant STGD3 protein in the retina reported different forms of rod and cone degeneration, including a transgenic pig model expressing the 5 bp del and the tyrosine premature stop (Y270terEYFP) (164) and *Xenopus laevis* (165). As we stated earlier, it seems that the absence of STGD3 protein in the retina of the conditional knockout animal models with depletion/loss of retinal VLC-PUFA leads to a slow and progressive retinal degeneration as seen in normal AMD (132, 133). What is puzzling is that expression of the STGD3 protein seems to induce early-onset retinal degeneration in STGD3 animal models (22, 167, 168, 169) and in humans (1, 2, 3, 4).

In other diseases, like Bardet-Biedl syndrome (BBS), a condition that leads to aberrant protein trafficking and blindness, the photoreceptor outer segments (POS) have an irregular packing pattern with large gaps that were described as large unusual vesicular structures (170, 171). This is similar to what was seen in TG2 ELOVL4 transgenic mice, where there were large gaps between disks of the OS (168). In the BBS mouse model, the leucine zipper transcription factor-like 1 (*Lztfl1*), a protein that interacts with BBS proteins to regulate protein trafficking, was knocked out (*Lztfl1*^−/−^) (170). In addition, in the BBS mice, more than half of photoreceptors were lost by 6 months of age. As a result, there was a significant reduction in both a-wave and b-wave in the *Lztfl1*^−/−^. In the BBS mouse models, proteins that would normally be targeted to the inner segment and outer plexiform layer were misrouted and accumulated in the POS (170). For example, in normal photoreceptor cells, STX3 and STXBP1 proteins, which are involved in membrane fusion, are localized to photoreceptor inner segment (IS) and the outer plexiform layer, with no detection in the OS. However, in *Lztfl1* mutant mice, both STX3 and STXBP1 proteins accumulate in the OS (171). The accumulation of non-OS proteins in POS is thought to be a causative factor in photoreceptor cell death and blindness in BBS. Based on these observations, the question remains whether the expression of the STGD3 protein and its mislocalization to OS are sufficient on their own to induce photoreceptor cell death. Thus, we cannot completely rule out the contribution of defective mutant ELOVL4, which lacks VLC-PUFA biosynthesis capability, on the development of macular degeneration in STGD3.

## Mislocalization of STGD3 in POS and effect on RPE function

At the cellular level, STGD3 protein is mislocalized away from the ER and resides in the cytoplasm in an aggregated pattern (16, 20, 21). Anatomically, Sommer *et al.* in 2011 reported mislocalization of the STGD3 mutant (Y270terEYFP) protein from the photoreceptor IS to the OS membrane (164). Furthermore, we showed that STGD3 protein is misrouted to the photoreceptor OS in *Xenopus laevis* models (165). Of interest, although replacing the lost ER retention sequence to the STGD3 protein restored its localization to the IS in the *Xenopus laevis* photoreceptors (165), it did not restore VLC-FA biosynthesis in cultured cells (29).

RPE cells continually phagocytose and degrade the distal end of POS that is essential for photoreceptor/RPE function and survival (172, 173, 174, 175, 176, 177, 178). Accumulation of undigested products in the RPE is a pathological feature of AMD (173, 179, 180). However, this phenotype is presented at a much younger age in some patients with STGD3 compared with patients with AMD. Therefore, there is the possibility that the presence of STGD3 protein in OS impairs the degradation machinery of RPE and may exert a toxic effect on RPE cells. Indeed, Kuny *et al.* (168) showed that the expression of the STGD3 mutant protein in photoreceptors exerts an increase in the accumulation of undigested POS. Ultrastructural examination of the outer segment surface with scanning electron microscopy, as well as analysis of cross-sections with transmission electron microscopy, revealed abnormal morphology of RPE starting at P30. By P90, TG1 and TG2 mice had large vacuoles in the RPE, which also had a less ordered ER, swollen intercristal spaces in the mitochondria, and large lysosomal deposits containing lipid and undigested OS prior to photoreceptor cell and vision loss (168). *In vitro* experiments also demonstrated that photoreceptors exert toxic effects on RPE cells (181, 182). When isolated POS from TG and WT littermate mice were fed to human RPE cells *in vitro*, the RPE cells showed a significant delay in digesting the outer segment membranes from the TG mice (181, 182). This delay in OS digestion interrupts the highly demanding and daily phagocytic activity by the RPE and, in turn, the recycling of materials necessary for photoreceptor health may be impaired. As a result, the accumulation of undigested products in STGD3 disease prior to photoreceptor degeneration indicates that the ineffective degradation in STGD3 OS plays a key role in RPE atrophy and subsequent photoreceptor dysfunction and death.

Mutations in ELOVL4 affect each of the ELOVL4-expressing tissues in a mutation-specific manner. There are several diseases in which the presence of a malfunctioning protein in the cell, regardless of its innate function, contributes to disease onset and progression (183, 184). It is therefore likely that the different tissue-specific disorders caused by the different ELOVL4 mutant proteins could be due to their specific structural alterations. Each group of mutations could affect how the mutant protein interacts with other fatty acid condensation and elongation enzymes, thereby leading to loss/depletion of specific tissue VLC-FA as a result of loss or alteration of enzymatic function. One plausible mechanism could be that the altered mutant ELOVL4 protein exerts a dominant negative effect on wild-type ELOVL4 and/or the entire fatty acid condensation-elongation machinery to affect cellular function or VLC-FA biosynthesis. On the other hand, we cannot rule out the possibility of the mutant ELOVL4 contributing to disease pathology through other cellular signaling mechanisms. Since ELOVL4 and other ELOVLs and their interacting partners are ER membrane-associated proteins that must work in concert to synthesize specific chain lengths of VLC-FA (77, 78, 185, 186, 187), mutations that affect one protein could affect the function of the entire lipid biosynthetic machinery.

Denic and Weissman (77) reported that, although the conserved (HXXHH) motif in the ELOVLs is not required for their incorporation into the multiprotein complexes necessary for global folding of the proteins, the motif is essential for the functional biosynthetic activity of the ELOVLs *in vivo*. Thus, considering the essential role of the histidine catalytic core and the ER retention/retrieval motifs in ELOVL4 enzymatic function, it is likely that ELOVL4 mutations that change the amino acid characteristics, and hence the structure of ELOVL4, close to the catalytic histidine core, or its transmembrane topology would affect the quality and quantity of VLC-FA synthesized in a specific tissue. Indeed, we showed that mutating any of the three histidine motifs affects VLC-FA biosynthesis (29). This finding suggests that structural integrity of ELOVL4 and probably the other ELOVL enzymes and their interacting partners is essential for their biological function.

## Conclusions and Future Directions

Although VLC-FA are essential lipids for life, they exist at low concentrations and are incorporated into a number of different lipid molecular species and structures that currently are difficult to identify. Thus, our current understanding of the biological function of VLC-FA-containing lipids is still limited. However, considering the importance of these fatty acids in life, there is a need for us to continue to investigate their enzymatic/molecular functions in order to understand their roles in different ELOVL4-expressing tissues. Considering the different tissue-specific disorders caused by the different ELOVL4 mutations, it is possible that mutations in ELOVL4 that do not substantially affect VLC-PUFA synthesis may not impact the levels of VLC-PUFA in the retina to an extent that would cause retinal degeneration as seen in patients with SCA34 who have no reported macular degeneration pathology. All of the SCA34-causing ELOVL4 mutations are full-length proteins possessing the essential ER retention/retrieval motif and histidine catalytic core that are crucial for VLC-FA biosynthesis and therefore could synthesize the VLC-PUFA needed for retinal function but not the fatty acids and lipids required for brain function. Also, a particular ELOVL4 mutation might interact with WT ELOVL4 protein in a way that specifically inhibits the ability of WT ELOVL4 to elongate VLC-SFA relative to VLC-PUFA, which may account for the different tissue-specific phenotypes presented by the different ELOVL4 mutations in patients. In other words, some mutations may significantly affect VLC-PUFA relative to VLC-SFA biosynthesis and therefore cause more severe disorders in one tissue relative to others. Compared with other tissues, the retina has relatively higher levels of VLC-PUFA. Therefore, it may take longer to deplete retinal VLC-PUFA to a level that would cause dysfunction relative to other ELOVL4-expressing tissues. This possibility may explain why SCA34 mutations may not cause STGD3.

VLC-PUFA-derived Elovanoids are important in mitigating cytotoxicity in photoreceptors (123, 159, 188). RPE atrophy is a hallmark of AMD and STGD3. RPE toxicity is seen prior to signs of photoreceptor loss in animal models (168, 169, 182). The retina and specifically photoreceptor cell survival depends on RPE's efficient phagocytosis of the distal tips of POS and the recycling of retinal outer segment components. The STGD3 mutations lead to a truncated protein that is mislocalized away from the ER and misrouted to the POS. Thus, the continuous delivery of the STGD3 protein to the OS and their subsequent uptake by the RPE may in the long term impair RPE function, and defective processing of POS materials by the RPE may contribute to RPE cytotoxicity. Since the STGD3 protein lacks VLC-PUFA biosynthesis, there are also the additional factors of age-related loss/depletion of VLC-PUFA and their Elovanoid derivatives that may serve essential signaling roles in upregulating prosurvival proteins to help compensate for any uncompensated oxidative stress in the RPE. At this stage, it is unclear what factors contribute to retinal and RPE defects in STGD3. However, based on the current evidence, it appears that photoreceptors VLC-PUFA deficits due to expression of STGD3 are detrimental to the RPE, leading to RPE toxicity and subsequent photoreceptor cell death. The decline in visual activity as measured by ERG in most STGD3 transgenic animal models may primarily be a result of photoreceptor dysfunction due to depletion of VLC-PUFA. Thus, photoreceptor-induced RPE toxicity may be a fundamental cause of STGD3.

In SCA34 and mutant ELOVL4-related seizures, we now understand that the enrichment of VLC-SFA in synaptic vesicles is important for brain function (68). Of interest, *Elovl4 Chx10-cKO* retinas had a reduction in synaptic vesicle number and diameter in rod presynaptic terminals and abnormal changes in synaptic function in the neural retina (133). These features suggest that, apart from VLC-PUFA, VLC-SFA may play a role in synaptic function in the retina. We also know that an age-dependent increase in methylation of the promoter region of *ELOVL2* is an underlying cause for decreases in biosynthesis of LC-PUFA, which in turn would affect the levels of tissue VLC-PUFA as we age. Thus, we hypothesize the following: (1) As we age, epigenetic modification of the regulatory and coding regions of genes essential for retinal lipid biosynthesis coupled with SNPs that cause decreased expression of lipid biosynthesis genes lead to an age-related decrease in essential retinal lipid levels; (2) The decrease in essential retinal lipids in turn affects lipid-mediated signaling pathways that are essential for retinal and RPE function and survival, leading to age-related vision loss; (3) When mutations such as defects in ELOVLs are involved, there are earlier retinal pathologies, as seen in patients with STGD3 and SCA34.

## Conflict of interest

The authors declare that they have no conflicts of interest with the contents of this article.

## References

[1] Zhang K., Kniazeva M., Han M., Li W., Yu Z., Yang Z., Li Y., Metzker M.L., Allikmets R., Zack D.J., Kakuk L.E., Lagali P.S., Wong P.W., MacDonald I.M. (2001). A 5-bp deletion in ELOVL4 is associated with two related forms of autosomal dominant macular dystrophy. Nat. Genet..

[2] Edwards A.O., Donoso L.A., Ritter R. (2001). A novel gene for autosomal dominant Stargardt-like macular dystrophy with homology to the SUR4 protein family. Invest. Ophthalmol. Vis. Sci..

[3] Bernstein P.S., Tammur J., Singh N., Hutchinson A., Dixon M., Pappas C.M., Zabriskie N.A., Zhang K., Petrukhin K., Leppert M., Allikmets R. (2001). Diverse macular dystrophy phenotype caused by a novel complex mutation in the ELOVL4 gene. Invest. Ophthalmol. Vis. Sci..

[4] Maugeri A., Meire F., Hoyng C.B., Vink C., Van Regemorter N., Karan G., Yang Z., Cremers F.P., Zhang K. (2004). A novel mutation in the ELOVL4 gene causes autosomal dominant Stargardt-like macular dystrophy. Invest. Ophthalmol. Vis. Sci..

[5] Ach T., Tolstik E., Messinger J.D., Zarubina A.V., Heintzmann R., Curcio C.A. (2015). Lipofuscin redistribution and loss accompanied by cytoskeletal stress in retinal pigment epithelium of eyes with age-related macular degeneration. Invest. Ophthalmol. Vis. Sci..

[6] Curcio C.A., Johnson M., Rudolf M., Huang J.D. (2011). The oil spill in ageing Bruch membrane. Br. J. Ophthalmol..

[7] Curcio C.A., Medeiros N.E., Millican C.L. (1996). Photoreceptor loss in age-related macular degeneration. Invest. Ophthalmol. Vis. Sci..

[8] Danis R.P., Lavine J.A., Domalpally A. (2015). Geographic atrophy in patients with advanced dry age-related macular degeneration: Current challenges and future prospects. Clin. Ophthalmol..

[9] Davis M.D., Gangnon R.E., Lee L.Y., Hubbard L.D., Klein B.E., Klein R., Ferris F.L., Bressler S.B., Milton R.C., Age-Related Eye Disease Study Group (2005). The Age-Related Eye Disease Study severity scale for age-related macular degeneration: AREDS Report No. 17. Arch. Ophthalmol..

[10] Donoso L.A., Edwards A.O., Frost A., Vrabec T., Stone E.M., Hageman G.S., Perski T. (2001). Autosomal dominant Stargardt-like macular dystrophy. Surv. Ophthalmol..

[11] Edwards A.O., Miedziak A., Vrabec T., Verhoeven J., Acott T.S., Weleber R.G., Donoso L.A. (1999). Autosomal dominant Stargardt-like macular dystrophy: I. Clinical characterization, longitudinal follow-up, and evidence for a common ancestry in families linked to chromosome 6q14. Am. J. Ophthalmol..

[12] Zhang X.M., Yang Z., Karan G., Hashimoto T., Baehr W., Yang X.J., Zhang K. (2003). Elovl4 mRNA distribution in the developing mouse retina and phylogenetic conservation of Elovl4 genes. Mol. Vis..

[13] Oh C.S., Toke D.A., Mandala S., Martin C.E. (1997). ELO2 and ELO3, homologues of the Saccharomyces cerevisiae ELO1 gene, function in fatty acid elongation and are required for sphingolipid formation. J. Biol. Chem..

[14] Paul S., Gable K., Beaudoin F., Cahoon E., Jaworski J., Napier J.A., Dunn T.M. (2006). Members of the Arabidopsis FAE1-like 3-ketoacyl-CoA synthase gene family substitute for the Elop proteins of Saccharomyces cerevisiae. J. Biol. Chem..

[15] Tvrdik P., Westerberg R., Silve S., Asadi A., Jakobsson A., Cannon B., Loison G., Jacobsson A. (2000). Role of a new mammalian gene family in the biosynthesis of very long chain fatty acids and sphingolipids. J. Cell Biol.

[16] Ambasudhan R., Wang X., Jablonski M.M., Thompson D.A., Lagali P.S., Wong P.W., Sieving P.A., Ayyagari R. (2004). Atrophic macular degeneration mutations in ELOVL4 result in the intracellular misrouting of the protein. Genomics.

[17] Karan G., Yang Z., Howes K., Zhao Y., Chen Y., Cameron D.J., Lin Y., Pearson E., Zhang K. (2005). Loss of ER retention and sequestration of the wild-type ELOVL4 by Stargardt disease dominant negative mutants. Mol. Vis..

[18] Karan G., Yang Z., Zhang K. (2004). Expression of wild type and mutant ELOVL4 in cell culture: Subcellular localization and cell viability. Mol. Vis..

[19] Vasireddy V., Jablonski M.M., Khan N.W., Wang X.F., Sahu P., Sparrow J.R., Ayyagari R. (2009). Elovl4 5-bp deletion knock-in mouse model for Stargardt-like macular degeneration demonstrates accumulation of ELOVL4 and lipofuscin. Exp. Eye Res..

[20] Vasireddy V., Vijayasarathy C., Huang J., Wang X.F., Jablonski M.M., Petty H.R., Sieving P.A., Ayyagari R. (2005). Stargardt-like macular dystrophy protein ELOVL4 exerts a dominant negative effect by recruiting wild-type protein into aggresomes. Mol. Vis..

[21] Grayson C., Molday R.S. (2005). Dominant negative mechanism underlies autosomal dominant Stargardt-like macular dystrophy linked to mutations in ELOVL4. J. Biol. Chem..

[22] Karan G., Lillo C., Yang Z., Cameron D.J., Locke K.G., Zhao Y., Thirumalaichary S., Li C., Birch D.G., Vollmer-Snarr H.R., Williams D.S., Zhang K. (2005). Lipofuscin accumulation, abnormal electrophysiology, and photoreceptor degeneration in mutant ELOVL4 transgenic mice: A model for macular degeneration. Proc. Natl. Acad. Sci. U. S. A..

[23] McMahon A., Butovich I.A., Mata N.L., Klein M., Ritter R., Richardson J., Birch D.G., Edwards A.O., Kedzierski W. (2007). Retinal pathology and skin barrier defect in mice carrying a Stargardt disease-3 mutation in elongase of very long chain fatty acids-4. Mol. Vis..

[24] McMahon A., Jackson S.N., Woods A.S., Kedzierski W. (2007). A Stargardt disease-3 mutation in the mouse Elovl4 gene causes retinal deficiency of C32-C36 acyl phosphatidylcholines. FEBS Lett..

[25] Raz-Prag D., Ayyagari R., Fariss R.N., Mandal M.N., Vasireddy V., Majchrzak S., Webber A.L., Bush R.A., Salem N., Petrukhin K., Sieving P.A. (2006). Haploinsufficiency is not the key mechanism of pathogenesis in a heterozygous Elovl4 knockout mouse model of STGD3 disease. Invest. Ophthalmol. Vis. Sci..

[26] Vasireddy V., Jablonski M.M., Mandal M.N., Raz-Prag D., Wang X.F., Nizol L., Iannaccone A., Musch D.C., Bush R.A., Salem N., Sieving P.A., Ayyagari R. (2006). Elovl4 5-bp-deletion knock-in mice develop progressive photoreceptor degeneration. Invest. Ophthalmol. Vis. Sci..

[27] Agbaga M.P., Brush R.S., Mandal M.N., Elliott M.H., Al-Ubaidi M.R., Anderson R.E. (2010). Role of elovl4 protein in the biosynthesis of docosahexaenoic Acid. Adv. Exp. Med. Biol..

[28] Agbaga M.P., Brush R.S., Mandal M.N., Henry K., Elliott M.H., Anderson R.E. (2008). Role of Stargardt-3 macular dystrophy protein (ELOVL4) in the biosynthesis of very long chain fatty acids. Proc. Natl. Acad. Sci. U. S. A..

[29] Logan S., Agbaga M.P., Chan M.D., Brush R.S., Anderson R.E. (2014). Endoplasmic reticulum microenvironment and conserved histidines govern ELOVL4 fatty acid elongase activity. J. Lipid Res..

[30] Logan S., Agbaga M.P., Chan M.D., Kabir N., Mandal N.A., Brush R.S., Anderson R.E. (2013). Deciphering mutant ELOVL4 activity in autosomal-dominant Stargardt macular dystrophy. Proc. Natl. Acad. Sci. U. S. A..

[31] Vasireddy V., Uchida Y., Salem N., Kim S.Y., Mandal M.N., Reddy G.B., Bodepudi R., Alderson N.L., Brown J.C., Hama H., Dlugosz A., Elias P.M., Holleran W.M., Ayyagari R. (2007). Loss of functional ELOVL4 depletes very long-chain fatty acids (> or =C28) and the unique omega-O-acylceramides in skin leading to neonatal death. Hum. Mol. Genet..

[32] Cameron D.J., Tong Z., Yang Z., Kaminoh J., Kamiyah S., Chen H., Zeng J., Chen Y., Luo L., Zhang K. (2007). Essential role of Elovl4 in very long chain fatty acid synthesis, skin permeability barrier function, and neonatal survival. Int. J. Biol. Sci..

[33] Li W., Sandhoff R., Kono M., Zerfas P., Hoffmann V., Ding B.C., Proia R.L., Deng C.X. (2007). Depletion of ceramides with very long chain fatty acids causes defective skin permeability barrier function, and neonatal lethality in ELOVL4 deficient mice. Int. J. Biol. Sci..

[34] Donato L., Scimone C., Rinaldi C., Aragona P., Briuglia S., D'Ascola A., D'Angelo R., Sidoti A. (2018). Stargardt phenotype associated with two ELOVL4 promoter variants and ELOVL4 downregulation: New possible perspective to etiopathogenesis?. Invest. Ophthalmol. Vis. Sci..

[35] Cadieux-Dion M., Turcotte-Gauthier M., Noreau A., Martin C., Meloche C., Gravel M., Drouin C.A., Rouleau G.A., Nguyen D.K., Cossette P. (2014). Expanding the clinical Phenotype associated with ELOVL4 mutation: Study of a Large French-Canadian family with autosomal dominant spinocerebellar ataxia and erythrokeratodermia. JAMA Neurol..

[36] Ozaki K., Doi H., Mitsui J., Sato N., Iikuni Y., Majima T., Yamane K., Irioka T., Ishiura H., Doi K., Morishita S., Higashi M., Sekiguchi T., Koyama K., Ueda N. (2015). A novel mutation in ELOVL4 leading to spinocerebellar ataxia (SCA) with the hot cross bun sign but lacking erythrokeratodermia: A broadened spectrum of SCA34. JAMA Neurol..

[37] Bourassa C.V., Raskin S., Serafini S., Teive H.A., Dion P.A., Rouleau G.A. (2015). A New ELOVL4 mutation in a case of spinocerebellar ataxia with erythrokeratodermia. JAMA Neurol..

[38] Bourque P.R., Warman-Chardon J., Lelli D.A., LaBerge L., Kirshen C., Bradshaw S.H., Hartley T., Boycott K.M. (2018). Novel ELOVL4 mutation associated with erythrokeratodermia and spinocerebellar ataxia (SCA 34). Neurol. Genet..

[39] Xiao C., M. B. E, Rexach J., Knight-Johnson A., Khemani P., Fogel B.L., Das S., Stone E.M., Gomez C.M. (2019). A family with spinocerebellar ataxia and retinitis pigmentosa attributed to an ELOVL4 mutation. Neurol. Genet..

[40] Aldahmesh M.A., Mohamed J.Y., Alkuraya H.S., Verma I.C., Puri R.D., Alaiya A.A., Rizzo W.B., Alkuraya F.S. (2011). Recessive mutations in ELOVL4 cause ichthyosis, intellectual disability, and spastic quadriplegia. Am. J. Hum. Genet..

[41] Mir H., Raza S.I., Touseef M., Memon M.M., Khan M.N., Jaffar S., Ahmad W. (2014). A novel recessive mutation in the gene ELOVL4 causes a neuro-ichthyotic disorder with variable expressivity. BMC Med. Genet..

[42] Agbaga M.P. (2016). Different mutations in ELOVL4 affect very long chain fatty acid biosynthesis to cause variable neurological disorders in humans. Adv. Exp. Med. Biol..

[43] Beaudin M., Sellami L., Martel C., Touzel-Deschenes L., Houle G., Martineau L., Lacroix K., Lavallee A., Chrestian N., Rouleau G.A., Gros-Louis F., Laforce R., Dupre N. (2020). Characterization of the phenotype with cognitive impairment and protein mislocalization in SCA34. Neurol. Genet..

[44] Allikmets R., Shroyer N.F., Singh N., Seddon J.M., Lewis R.A., Bernstein P.S., Peiffer A., Zabriskie N.A., Li Y., Hutchinson A., Dean M., Lupski J.R., Leppert M. (1997). Mutation of the Stargardt disease gene (ABCR) in age-related macular degeneration. Science.

[45] Molday L.L., Rabin A.R., Molday R.S. (2000). ABCR expression in foveal cone photoreceptors and its role in Stargardt macular dystrophy. Nat. Genet..

[46] Maugeri A., Klevering B.J., Rohrschneider K., Blankenagel A., Brunner H.G., Deutman A.F., Hoyng C.B., Cremers F.P. (2000). Mutations in the ABCA4 (ABCR) gene are the major cause of autosomal recessive cone-rod dystrophy. Am. J. Hum. Genet..

[47] Zhang K., Garibaldi D.C., Kniazeva M., Albini T., Chiang M.F., Kerrigan M., Sunness J.S., Han M., Allikmets R. (1999). A novel mutation in the ABCR gene in four patients with autosomal recessive Stargardt disease. Am. J. Ophthalmol..

[48] Allikmets R., Singh N., Sun H., Shroyer N.F., Hutchinson A., Chidambaram A., Gerrard B., Baird L., Stauffer D., Peiffer A., Rattner A., Smallwood P., Li Y., Anderson K.L., Lewis R.A. (1997). A photoreceptor cell-specific ATP-binding transporter gene (ABCR) is mutated in recessive Stargardt macular dystrophy. Nat. Genet..

[49] Arnell H., Mantyjarvi M., Tuppurainen K., Andreasson S., Dahl N. (1998). Stargardt disease: Linkage to the ABCR gene region on 1p21-p22 in Scandinavian families. Acta Ophthalmol. Scand..

[50] Sun H., Nathans J. (1997). Stargardt's ABCR is localized to the disc membrane of retinal rod outer segments. Nat. Genet..

[51] Eksandh L., Ekstrom U., Abrahamson M., Bauer B., Andreasson S. (2001). Different clinical expressions in two families with Stargardt's macular dystrophy (STGD1). Acta Ophthalmol. Scand..

[52] Zhang K., Kniazeva M., Hutchinson A., Han M., Dean M., Allikmets R. (1999). The ABCR gene in recessive and dominant Stargardt diseases: A genetic pathway in macular degeneration. Genomics.

[53] Kniazeva M., Chiang M.F., Morgan B., Anduze A.L., Zack D.J., Han M., Zhang K. (1999). A new locus for autosomal dominant stargardt-like disease maps to chromosome 4. Am. J. Hum. Genet..

[54] Quazi F., Molday R.S. (2013). Differential phospholipid substrates and directional transport by ATP-binding cassette proteins ABCA1, ABCA7, and ABCA4 and disease-causing mutants. J. Biol. Chem..

[55] Radu R.A., Mata N.L., Bagla A., Travis G.H. (2004). Light exposure stimulates formation of A2E oxiranes in a mouse model of Stargardt's macular degeneration. Proc. Natl. Acad. Sci. U. S. A..

[56] Kim H.J., Zhao J., Sparrow J.R. (2020). Vitamin A aldehyde-taurine adduct and the visual cycle. Proc. Natl. Acad. Sci. U. S. A..

[57] Green J.S., O'Rielly D.D., Pater J.A., Houston J., Rajabi H., Galutira D., Benteau T., Sheaves A., Abdelfatah N., Bautista D., Whelan J., Young T.L. (2020). The genetic architecture of Stargardt macular dystrophy (STGD1): A longitudinal 40-year study in a genetic isolate. Eur. J. Hum. Genet..

[58] Michaelides M., Hunt D.M., Moore A.T. (2003). The genetics of inherited macular dystrophies. J. Med. Genet..

[59] Conley S.M., Cai X., Makkia R., Wu Y., Sparrow J.R., Naash M.I. (2012). Increased cone sensitivity to ABCA4 deficiency provides insight into macular vision loss in Stargardt's dystrophy. Biochim. Biophys. Acta.

[60] Rudolph G., Kalpadakis P., Haritoglou C., Rivera A., Weber B.H. (2002). Klin. Monbl. Augenheilkd.

[61] Hu F., Gao F., Li J., Xu P., Wang D., Chen F., Zhang S., Wu J. (2020). Novel variants associated with Stargardt disease in Chinese patients. Gene.

[62] Zhong M., Molday L.L., Molday R.S. (2009). Role of the C terminus of the photoreceptor ABCA4 transporter in protein folding, function, and retinal degenerative diseases. J. Biol. Chem..

[63] Molday R.S., Zhong M., Quazi F. (2009). The role of the photoreceptor ABC transporter ABCA4 in lipid transport and Stargardt macular degeneration. Biochim. Biophys. Acta.

[64] Bardak H., Gunay M., Ercalik Y., Bardak Y., Ozbas H., Bagci O., Ayata A., Sonmez M., Alagoz C. (2016). Analysis of ELOVL4 and PRPH2 genes in Turkish Stargardt disease patients. Genet. Mol. Res..

[65] Ozaki K., Ansai A., Nobuhara K., Araki T., Kubodera T., Ishii T., Higashi M., Sato N., Soga K., Mizusawa H., Ishikawa K., Yokota T. (2019). Prevalence and clinicoradiological features of spinocerebellar ataxia type 34 in a Japanese ataxia cohort. Parkinsonism Relat. Disord..

[66] Donoso L.A., Frost A.T., Stone E.M., Weleber R.G., MacDonald I.M., Hageman G.S., Cibis G.W., Ritter R., Edwards A.O. (2001). Autosomal dominant Stargardt-like macular dystrophy: Founder effect and reassessment of genetic heterogeneity. Arch. Ophthalmol..

[67] McMahon A., Butovich I.A., Kedzierski W. (2011). Epidermal expression of an Elovl4 transgene rescues neonatal lethality of homozygous Stargardt disease-3 mice. J. Lipid Res..

[68] Hopiavuori B.R., Deak F., Wilkerson J.L., Brush R.S., Rocha-Hopiavuori N.A., Hopiavuori A.R., Ozan K.G., Sullivan M.T., Wren J.D., Georgescu C., Szweda L., Awasthi V., Towner R., Sherry D.M., Anderson R.E. (2018). Homozygous expression of mutant ELOVL4 leads to seizures and death in a novel animal model of very long-chain fatty acid deficiency. Mol. Neurobiol..

[69] Yu M., Benham A., Logan S., Brush R.S., Mandal M.N., Anderson R.E., Agbaga M.P. (2012). ELOVL4 protein preferentially elongates 20:5n3 to very long chain PUFAs over 20:4n6 and 22:6n3. J. Lipid Res..

[70] Hopiavuori B.R., Anderson R.E., Agbaga M.P. (2019). ELOVL4: Very long-chain fatty acids serve an eclectic role in mammalian health and function. Prog. Retin. Eye Res..

[71] Jakobsson A., Westerberg R., Jacobsson A. (2006). Fatty acid elongases in mammals: Their regulation and roles in metabolism. Prog. Lipid Res..

[72] Guillou H., Zadravec D., Martin P.G., Jacobsson A. (2010). The key roles of elongases and desaturases in mammalian fatty acid metabolism: Insights from transgenic mice. Prog. Lipid Res..

[73] Ohno Y., Suto S., Yamanaka M., Mizutani Y., Mitsutake S., Igarashi Y., Sassa T., Kihara A. (2010). ELOVL1 production of C24 acyl-CoAs is linked to C24 sphingolipid synthesis. Proc. Natl. Acad. Sci. U. S. A..

[74] Bedell M., Harkewicz R., Wang X., Zhang K. (2010). Focus on molecules: ELOVL4. Exp. Eye Res..

[75] Jump D.B. (2009). Mammalian fatty acid elongases. Methods Mol. Biol..

[76] Okuda A., Naganuma T., Ohno Y., Abe K., Yamagata M., Igarashi Y., Kihara A. (2010). Hetero-oligomeric interactions of an ELOVL4 mutant protein: Implications in the molecular mechanism of Stargardt-3 macular dystrophy. Mol. Vis..

[77] Denic V., Weissman J.S. (2007). A molecular caliper mechanism for determining very long-chain fatty acid length. Cell.

[78] Riezman H. (2007). The long and short of fatty acid synthesis. Cell.

[79] Logan S., Anderson R.E. (2014). Dominant Stargardt Macular Dystrophy (STGD3) and ELOVL4. Adv. Exp. Med. Biol..

[80] Mandal M.N., Ambasudhan R., Wong P.W., Gage P.J., Sieving P.A., Ayyagari R. (2004). Characterization of mouse orthologue of ELOVL4: Genomic organization and spatial and temporal expression. Genomics.

[81] McMahon A., Lu H., Butovich I.A. (2014). A role for ELOVL4 in the mouse Meibomian gland and sebocyte cell biology. Invest. Ophthalmol. Vis. Sci..

[82] Aveldano M.I. (1987). A novel group of very long chain polyenoic fatty acids in dipolyunsaturated phosphatidylcholines from vertebrate retina. J. Biol. Chem..

[83] Aveldano M.I., Sprecher H. (1987). Very long chain (C24 to C36) polyenoic fatty acids of the n-3 and n-6 series in dipolyunsaturated phosphatidylcholines from bovine retina. J. Biol. Chem..

[84] Aveldano M.I. (1988). Phospholipid species containing long and very long polyenoic fatty acids remain with rhodopsin after hexane extraction of photoreceptor membranes. Biochemistry.

[85] Lagali P.S., Liu J., Ambasudhan R., Kakuk L.E., Bernstein S.L., Seigel G.M., Wong P.W., Ayyagari R. (2003). Evolutionarily conserved ELOVL4 gene expression in the vertebrate retina. Invest. Ophthalmol. Vis. Sci..

[86] Umeda S., Ayyagari R., Suzuki M.T., Ono F., Iwata F., Fujiki K., Kanai A., Takada Y., Yoshikawa Y., Tanaka Y., Iwata T. (2003). Molecular cloning of ELOVL4 gene from cynomolgus monkey (Macaca fascicularis). Exp. Anim..

[87] Butovich I.A. (2008). On the lipid composition of human meibum and tears: Comparative analysis of nonpolar lipids. Invest. Ophthalmol. Vis. Sci..

[88] Butovich I.A. (2009). Lipidomic analysis of human meibum using HPLC-MSn. Methods Mol. Biol..

[89] Butovich I.A. (2009). The Meibomian puzzle: Combining pieces together. Prog. Retin. Eye Res..

[90] Butovich I.A. (2009). Cholesteryl esters as a depot for very long chain fatty acids in human meibum. J. Lipid Res..

[91] Butovich I.A., Uchiyama E., McCulley J.P. (2007). Lipids of human meibum: Mass-spectrometric analysis and structural elucidation. J. Lipid Res..

[92] Butovich I.A., Wojtowicz J.C., Molai M. (2009). Human tear film and meibum. Very long chain wax esters and (O-acyl)-omega-hydroxy fatty acids of meibum. J. Lipid Res..

[93] Joffre C., Souchier M., Gregoire S., Viau S., Bretillon L., Acar N., Bron A.M., Creuzot-Garcher C. (2008). Differences in meibomian fatty acid composition in patients with meibomian gland dysfunction and aqueous-deficient dry eye. Br. J. Ophthalmol..

[94] Sherry D.M., Hopiavuori B.R., Stiles M.A., Rahman N.S., Ozan K.G., Deak F., Agbaga M.P., Anderson R.E. (2017). Distribution of ELOVL4 in the developing and adult mouse brain. Front. Neuroanat..

[95] Uchida Y., Holleran W.M. (2008). Omega-O-acylceramide, a lipid essential for mammalian survival. J. Dermatol. Sci..

[96] Kitajka K., Sinclair A.J., Weisinger R.S., Weisinger H.S., Mathai M., Jayasooriya A.P., Halver J.E., Puskas L.G. (2004). Effects of dietary omega-3 polyunsaturated fatty acids on brain gene expression. Proc. Natl. Acad. Sci. U. S. A..

[97] Alessandri J.M., Guesnet P., Vancassel S., Astorg P., Denis I., Langelier B., Aid S., Poumes-Ballihaut C., Champeil-Potokar G., Lavialle M. (2004). Polyunsaturated fatty acids in the central nervous system: Evolution of concepts and nutritional implications throughout life. Reprod. Nutr. Dev..

[98] Alessandri J.M., Poumes-Ballihaut C., Langelier B., Perruchot M.H., Raguenez G., Lavialle M., Guesnet P. (2003). Incorporation of docosahexaenoic acid into nerve membrane phospholipids: Bridging the gap between animals and cultured cells. Am. J. Clin. Nutr..

[99] Anderson G.J., Neuringer M., Lin D.S., Connor W.E. (2005). Can prenatal N-3 fatty acid deficiency be completely reversed after birth? Effects on retinal and brain biochemistry and visual function in rhesus monkeys. Pediatr. Res..

[100] Bazan N.G. (2007). Omega-3 fatty acids, pro-inflammatory signaling and neuroprotection. Curr. Opin. Clin. Nutr. Metab. Care.

[101] Carlson S.E. (2001). Docosahexaenoic acid and arachidonic acid in infant development. Semin. Neonatol.

[102] Freemantle E., Vandal M., Tremblay-Mercier J., Tremblay S., Blachere J.C., Begin M.E., Brenna J.T., Windust A., Cunnane S.C. (2006). Omega-3 fatty acids, energy substrates, and brain function during aging. Prostaglandins Leukot. Essent. Fatty Acids.

[103] Greiner R.C., Winter J., Nathanielsz P.W., Brenna J.T. (1997). Brain docosahexaenoate accretion in fetal baboons: Bioequivalence of dietary alpha-linolenic and docosahexaenoic acids. Pediatr. Res..

[104] Kaduce T.L., Chen Y., Hell J.W., Spector A.A. (2008). Docosahexaenoic acid synthesis from n-3 fatty acid precursors in rat hippocampal neurons. J. Neurochem..

[105] Levant B., Ozias M.K., Carlson S.E. (2007). Specific brain regions of female rats are differentially depleted of docosahexaenoic acid by reproductive activity and an (n-3) fatty acid-deficient diet. J. Nutr..

[106] Brush R.S., Tran J.T., Henry K.R., McClellan M.E., Elliott M.H., Mandal M.N. (2010). Retinal sphingolipids and their very-long-chain fatty acid-containing species. Invest. Ophthalmol. Vis. Sci..

[107] Aveldano M.I. (1992). Long and very long polyunsaturated fatty acids of retina and spermatozoa: The whole complement of polyenoic fatty acid series. Adv. Exp. Med. Biol..

[108] Craig L.B., Brush R.S., Sullivan M.T., Zavy M.T., Agbaga M.P., Anderson R.E. (2019). Decreased very long chain polyunsaturated fatty acids in sperm correlates with sperm quantity and quality. J. Assist Reprod. Genet..

[109] Roqueta-Rivera M., Stroud C.K., Haschek W.M., Akare S.J., Segre M., Brush R.S., Agbaga M.P., Anderson R.E., Hess R.A., Nakamura M.T. (2010). Docosahexaenoic acid supplementation fully restores fertility and spermatogenesis in male delta-6 desaturase-null mice. J. Lipid Res..

[110] Poulos A., Christodoulou J., Chow C.W., Goldblatt J., Paton B.C., Orii T., Suzuki Y., Shimozawa N. (1995). Peroxisomal assembly defects: Clinical, pathologic, and biochemical findings in two patients in a newly identified complementation group. J. Pediatr..

[111] Poulos A., Sharp P., Johnson D. (1989). Plasma polyenoic very-long-chain fatty acids in peroxisomal disease: Biochemical discrimination of Zellweger's syndrome from other phenotypes. Neurology.

[112] Poulos A., Sharp P., Johnson D., Easton C. (1988). The occurrence of polyenoic very long chain fatty acids with greater than 32 carbon atoms in molecular species of phosphatidylcholine in normal and peroxisome-deficient (Zellweger's syndrome) brain. Biochem. J..

[113] Madsen L., Rustan A.C., Vaagenes H., Berge K., Dyroy E., Berge R.K. (1999). Eicosapentaenoic and docosahexaenoic acid affect mitochondrial and peroxisomal fatty acid oxidation in relation to substrate preference. Lipids.

[114] Madsen L., Froyland L., Dyroy E., Helland K., Berge R.K. (1998). Docosahexaenoic and eicosapentaenoic acids are differently metabolized in rat liver during mitochondria and peroxisome proliferation. J. Lipid Res..

[115] Lazarow P.B. (1978). Rat liver peroxisomes catalyze the beta oxidation of fatty acids. J. Biol. Chem..

[116] Chen C.T., Liu Z., Ouellet M., Calon F., Bazinet R.P. (2009). Rapid beta-oxidation of eicosapentaenoic acid in mouse brain: An in situ study. Prostaglandins Leukot. Essent. Fatty Acids.

[117] Leyton J., Drury P.J., Crawford M.A. (1987). Differential oxidation of saturated and unsaturated fatty acids in vivo in the rat. Br. J. Nutr..

[118] Carmona-Antonanzas G., Monroig O., Dick J.R., Davie A., Tocher D.R. (2011). Biosynthesis of very long-chain fatty acids (C>24) in Atlantic salmon: Cloning, functional characterisation, and tissue distribution of an Elovl4 elongase. Comp. Biochem. Physiol. B Biochem. Mol. Biol..

[119] Zemski Berry K.A., Gordon W.C., Murphy R.C., Bazan N.G. (2014). Spatial organization of lipids in the human retina and optic nerve by MALDI imaging mass spectrometry. J. Lipid Res..

[120] Suh M., Clandinin M.T. (2005). 20:5n-3 but not 22:6n-3 is a preferred substrate for synthesis of n-3 very-long- chain fatty acids (C24-C36) in retina. Curr. Eye Res..

[121] Stillwell W., Wassall S.R. (2003). Docosahexaenoic acid: Membrane properties of a unique fatty acid. Chem. Phys. Lipids.

[122] Lauritzen L., Hansen H.S., Jorgensen M.H., Michaelsen K.F. (2001). The essentiality of long chain n-3 fatty acids in relation to development and function of the brain and retina. Prog. Lipid Res..

[123] Bhattacharjee S., Jun B., Belayev L., Heap J., Kautzmann M.A., Obenaus A., Menghani H., Marcell S.J., Khoutorova L., Yang R., Petasis N.A., Bazan N.G. (2017). Elovanoids are a novel class of homeostatic lipid mediators that protect neural cell integrity upon injury. Sci. Adv..

[124] Rice D.S., Calandria J.M., Gordon W.C., Jun B., Zhou Y., Gelfman C.M., Li S., Jin M., Knott E.J., Chang B., Abuin A., Issa T., Potter D., Platt K.A., Bazan N.G. (2015). Adiponectin receptor 1 conserves docosahexaenoic acid and promotes photoreceptor cell survival. Nat. Commun..

[125] Mohrhauer H., Christiansen K., Gan M.V., Deubig M., Holman R.T. (1967). Chain elongation of linoleic acid and its inhibition by other fatty acids in vitro. J. Biol. Chem..

[126] Burns T.A., Duckett S.K., Pratt S.L., Jenkins T.C. (2012). Supplemental palmitoleic (C16:1 cis-9) acid reduces lipogenesis and desaturation in bovine adipocyte cultures. J. Anim. Sci..

[127] Gregory M.K., Cleland L.G., James M.J. (2013). Molecular basis for differential elongation of omega-3 docosapentaenoic acid by the rat Elovl5 and Elovl2. J. Lipid Res..

[128] Gregory M.K., James M.J. (2014). Functional characterization of the duck and turkey fatty acyl elongase enzymes ELOVL5 and ELOVL2. J. Nutr..

[129] Agbaga M.P., Stiles M.A., Brush R.S., Sullivan M.T., Machalinski A., Jones K.L., Anderson R.E., Sherry D.M. (2020). The Elovl4 spinocerebellar Ataxia-34 mutation 736T>G (p.W246G) impairs retinal function in the absence of photoreceptor degeneration. Mol. Neurobiol..

[130] Parisi L.R., Sowlati-Hashjin S., Berhane I.A., Galster S.L., Carter K.A., Lovell J.F., Chemler S.R., Karttunen M., Atilla-Gokcumen G.E. (2019). Membrane disruption by very long chain fatty acids during necroptosis. ACS Chem. Biol..

[131] Parisi L.R., Li N., Atilla-Gokcumen G.E. (2017). Very long chain fatty acids are functionally involved in necroptosis. Cell Chem Biol.

[132] Bennett L.D., Brush R.S., Chan M., Lydic T.A., Reese K., Reid G.E., Busik J.V., Elliott M.H., Anderson R.E. (2014). Effect of reduced retinal VLC-PUFA on rod and cone photoreceptors. Invest. Ophthalmol. Vis. Sci..

[133] Bennett L.D., Hopiavuori B.R., Brush R.S., Chan M., Van Hook M.J., Thoreson W.B., Anderson R.E. (2014). Examination of VLC-PUFA-deficient photoreceptor terminals. Invest. Ophthalmol. Vis. Sci..

[134] Harkewicz R., Du H., Tong Z., Alkuraya H., Bedell M., Sun W., Wang X., Hsu Y.H., Esteve-Rudd J., Hughes G., Su Z., Zhang M., Lopes V.S., Molday R.S., Williams D.S. (2012). Essential role of ELOVL4 protein in very long chain fatty acid synthesis and retinal function. J. Biol. Chem..

[135] Liu A., Chang J., Lin Y., Shen Z., Bernstein P.S. (2010). Long-chain and very long-chain polyunsaturated fatty acids in ocular aging and age-related macular degeneration. J. Lipid Res..

[136] Gorusupudi A., Liu A., Hageman G.S., Bernstein P.S. (2016). Associations of human retinal very long-chain polyunsaturated fatty acids with dietary lipid biomarkers. J. Lipid Res..

[137] Ayyagari R., Zhang K., Hutchinson A., Yu Z., Swaroop A., Kakuk L.E., Seddon J.M., Bernstein P.S., Lewis R.A., Tammur J., Yang Z., Li Y., Zhang H., Yashar B.M., Liu J. (2001). Evaluation of the ELOVL4 gene in patients with age-related macular degeneration. Ophthalmic Genet..

[138] DeAngelis M.M., Ji F., Kim I.K., Adams S., Capone A., Ott J., Miller J.W., Dryja T.P. (2007). Cigarette smoking, CFH, APOE, ELOVL4, and risk of neovascular age-related macular degeneration. Arch. Ophthalmol..

[139] Li X., Wang J., Wang L., Feng G., Li G., Yu M., Li Y., Liu C., Yuan X., Zang G., Li Z., Zhao L., Ouyang H., Quan Q., Wang G. (2020). Impaired lipid metabolism by age-dependent DNA methylation alterations accelerates aging. Proc. Natl. Acad. Sci. U. S. A..

[140] Chen D., Chao D.L., Rocha L., Kolar M., Nguyen Huu V.A., Krawczyk M., Dasyani M., Wang T., Jafari M., Jabari M., Ross K.D., Saghatelian A., Hamilton B.A., Zhang K., Skowronska-Krawczyk D. (2020). The lipid elongation enzyme ELOVL2 is a molecular regulator of aging in the retina. Aging cell.

[141] Garagnani P., Bacalini M.G., Pirazzini C., Gori D., Giuliani C., Mari D., Di Blasio A.M., Gentilini D., Vitale G., Collino S., Rezzi S., Castellani G., Capri M., Salvioli S., Franceschi C. (2012). Methylation of ELOVL2 gene as a new epigenetic marker of age. Aging Cell.

[142] Kananen L., Marttila S., Nevalainen T., Jylhava J., Mononen N., Kahonen M., Raitakari O.T., Lehtimaki T., Hurme M. (2016). Aging-associated DNA methylation changes in middle-aged individuals: The Young Finns study. BMC Genomics.

[143] Alsaleh A., Maniou Z., Lewis F.J., Hall W.L., Sanders T.A., O'Dell S.D. (2014). ELOVL2 gene polymorphisms are associated with increases in plasma eicosapentaenoic and docosahexaenoic acid proportions after fish oil supplement. Genes Nutr..

[144] Birch D.G., Anderson J.L. (1992). Standardized full-field electroretinography. Normal values and their variation with age. Arch. Ophthalmol..

[145] Birch D.G., Birch E.E., Hoffman D.R., Uauy R.D. (1992). Retinal development in very-low-birth-weight infants fed diets differing in omega-3 fatty acids. Invest. Ophthalmol. Vis. Sci..

[146] Eagle R.C., Lucier A.C., Bernardino V.B., Yanoff M. (1980). Retinal pigment epithelial abnormalities in fundus flavimaculatus: A light and electron microscopic study. Ophthalmology.

[147] Finnemann S.C., Leung L.W., Rodriguez-Boulan E. (2002). The lipofuscin component A2E selectively inhibits phagolysosomal degradation of photoreceptor phospholipid by the retinal pigment epithelium. Proc. Natl. Acad. Sci. U. S. A..

[148] Marmorstein A.D., Marmorstein L.Y., Sakaguchi H., Hollyfield J.G. (2002). Spectral profiling of autofluorescence associated with lipofuscin, Bruch's Membrane, and sub-RPE deposits in normal and AMD eyes. Invest. Ophthalmol. Vis. Sci..

[149] Zanzottera E.C., Ach T., Huisingh C., Messinger J.D., Freund K.B., Curcio C.A. (2016). Visualizing retinal pigment epithelium phenotypes in the transition to atrophy in neovascular age-related macular degeneration. Retina.

[150] Li W., Chen Y., Cameron D.J., Wang C., Karan G., Yang Z., Zhao Y., Pearson E., Chen H., Deng C., Howes K., Zhang K. (2007). Elovl4 haploinsufficiency does not induce early onset retinal degeneration in mice. Vis. Res.

[151] Agbaga M.P., Merriman D.K., Brush R.S., Lydic T.A., Conley S.M., Naash M.I., Jackson S., Woods A.S., Reid G.E., Busik J.V., Anderson R.E. (2018). Differential composition of DHA and very-long-chain PUFAs in rod and cone photoreceptors. J. Lipid Res..

[152] Schori C., Agbaga M.P., Brush R.S., Ayyagari R., Grimm C., Samardzija M. (2018). Elovl4 5-bp deletion does not accelerate cone photoreceptor degeneration in an all-cone mouse. PLoS One.

[153] van Kuijk F.J., Buck P. (1992). Fatty acid composition of the human macula and peripheral retina. Invest. Ophthalmol. Vis. Sci..

[154] Age-Related Eye Disease Study 2 Research G. (2013). Lutein + zeaxanthin and omega-3 fatty acids for age-related macular degeneration: The Age-Related Eye Disease Study 2 (AREDS2) randomized clinical trial. JAMA.

[155] Souied E.H., Aslam T., Garcia-Layana A., Holz F.G., Leys A., Silva R., Delcourt C. (2015). Omega-3 fatty acids and age-related macular degeneration. Ophthalmic Res..

[156] Beatty S., Koh H., Phil M., Henson D., Boulton M. (2000). The role of oxidative stress in the pathogenesis of age-related macular degeneration. Surv. Ophthalmol..

[157] Hao W., Wenzel A., Obin M.S., Chen C.K., Brill E., Krasnoperova N.V., Eversole-Cire P., Kleyner Y., Taylor A., Simon M.I., Grimm C., Reme C.E., Lem J. (2002). Evidence for two apoptotic pathways in light-induced retinal degeneration. Nat. Genet..

[158] Hunter J.J., Morgan J.I., Merigan W.H., Sliney D.H., Sparrow J.R., Williams D.R. (2012). The susceptibility of the retina to photochemical damage from visible light. Prog. Retin. Eye Res..

[159] Jun B., Mukherjee P.K., Asatryan A., Kautzmann M.A., Heap J., Gordon W.C., Bhattacharjee S., Yang R., Petasis N.A., Bazan N.G. (2017). Elovanoids are novel cell-specific lipid mediators necessary for neuroprotective signaling for photoreceptor cell integrity. Sci. Rep..

[160] Lobanova E.S., Schuhmann K., Finkelstein S., Lewis T.R., Cady M.A., Hao Y., Keuthan C., Ash J.D., Burns M.E., Shevchenko A., Arshavsky V.Y. (2019). Disrupted blood-retina lysophosphatidylcholine transport impairs photoreceptor health but not visual signal transduction. J. Neurosci..

[161] Nguyen L.N., Ma D., Shui G., Wong P., Cazenave-Gassiot A., Zhang X., Wenk M.R., Goh E.L., Silver D.L. (2014). Mfsd2a is a transporter for the essential omega-3 fatty acid docosahexaenoic acid. Nature.

[162] Wong B.H., Chan J.P., Cazenave-Gassiot A., Poh R.W., Foo J.C., Galam D.L., Ghosh S., Nguyen L.N., Barathi V.A., Yeo S.W., Luu C.D., Wenk M.R., Silver D.L. (2016). Mfsd2a is a transporter for the essential omega-3 fatty acid docosahexaenoic acid (DHA) in eye and is important for photoreceptor cell development. J. Biol. Chem..

[163] Chan J.P., Wong B.H., Chin C.F., Galam D.L.A., Foo J.C., Wong L.C., Ghosh S., Wenk M.R., Cazenave-Gassiot A., Silver D.L. (2018). The lysolipid transporter Mfsd2a regulates lipogenesis in the developing brain. PLoS Biol..

[164] Sommer J.R., Estrada J.L., Collins E.B., Bedell M., Alexander C.A., Yang Z., Hughes G., Mir B., Gilger B.C., Grob S., Wei X., Piedrahita J.A., Shaw P.X., Petters R.M., Zhang K. (2011). Production of ELOVL4 transgenic pigs: A large animal model for Stargardt-like macular degeneration. Br. J. Ophthalmol..

[165] Agbaga M.P., Tam B.M., Wong J.S., Yang L.L., Anderson R.E., Moritz O.L. (2014). Mutant ELOVL4 that causes autosomal dominant Stargardt-3 macular dystrophy is misrouted to rod outer segment disks. Invest. Ophthalmol. Vis. Sci..

[166] Mandal N.A., Tran J.T., Zheng L., Wilkerson J.L., Brush R.S., McRae J., Agbaga M.P., Zhang K., Petrukhin K., Ayyagari R., Anderson R.E. (2014). In vivo effect of mutant ELOVL4 on the expression and function of wild-type ELOVL4. Invest. Ophthalmol. Vis. Sci..

[167] Dornstauder B., Suh M., Kuny S., Gaillard F., Macdonald I.M., Clandinin M.T., Sauve Y. (2012). Dietary docosahexaenoic acid supplementation prevents age-related functional losses and A2E accumulation in the retina. Invest. Ophthalmol. Vis. Sci..

[168] Kuny S., Cho W.J., Dimopoulos I.S., Sauve Y. (2015). Early onset ultrastructural and functional defects in rpe and photoreceptors of a Stargardt-like macular dystrophy (STGD3) transgenic mouse model. Invest. Ophthalmol. Vis. Sci..

[169] Kuny S., Filion M.A., Suh M., Gaillard F., Sauve Y. (2014). Long-term retinal cone survival and delayed alteration of the cone mosaic in a transgenic mouse model of stargardt-like dystrophy (STGD3). Invest. Ophthalmol. Vis. Sci..

[170] Jiang J., Promchan K., Jiang H., Awasthi P., Marshall H., Harned A., Natarajan V. (2016). Depletion of BBS protein LZTFL1 affects growth and causes retinal degeneration in mice. J. Genet. Genomics.

[171] Datta P., Allamargot C., Hudson J.S., Andersen E.K., Bhattarai S., Drack A.V., Sheffield V.C., Seo S. (2015). Accumulation of non-outer segment proteins in the outer segment underlies photoreceptor degeneration in Bardet-Biedl syndrome. Proc. Natl. Acad. Sci. U. S. A..

[172] Feng W., Yasumura D., Matthes M.T., LaVail M.M., Vollrath D. (2002). Mertk triggers uptake of photoreceptor outer segments during phagocytosis by cultured retinal pigment epithelial cells. J. Biol. Chem..

[173] Ferrington D.A., Sinha D., Kaarniranta K. (2016). Defects in retinal pigment epithelial cell proteolysis and the pathology associated with age-related macular degeneration. Prog. Retin. Eye Res..

[174] Finnemann S.C. (2003). Role of alphavbeta5 integrin in regulating phagocytosis by the retinal pigment epithelium. Adv. Exp. Med. Biol..

[175] Finnemann S.C., Bonilha V.L., Marmorstein A.D., Rodriguez-Boulan E. (1997). Phagocytosis of rod outer segments by retinal pigment epithelial cells requires alpha(v)beta5 integrin for binding but not for internalization. Proc. Natl. Acad. Sci. U. S. A..

[176] LaVail M.M. (1973). Kinetics of rod outer segment renewal in the developing mouse retina. J. Cell Biol.

[177] LaVail M.M., Mullen R.J. (1976). Role of the pigment epithelium in inherited retinal degeneration analyzed with experimental mouse chimeras. Exp. Eye Res..

[178] Mustafi D., Kevany B.M., Genoud C., Okano K., Cideciyan A.V., Sumaroka A., Roman A.J., Jacobson S.G., Engel A., Adams M.D., Palczewski K. (2011). Defective photoreceptor phagocytosis in a mouse model of enhanced S-cone syndrome causes progressive retinal degeneration. FASEB J..

[179] Kaarniranta K., Sinha D., Blasiak J., Kauppinen A., Vereb Z., Salminen A., Boulton M.E., Petrovski G. (2013). Autophagy and heterophagy dysregulation leads to retinal pigment epithelium dysfunction and development of age-related macular degeneration. Autophagy.

[180] Sinha D., Valapala M., Shang P., Hose S., Grebe R., Lutty G.A., Zigler J.S., Kaarniranta K., Handa J.T. (2016). Lysosomes: Regulators of autophagy in the retinal pigmented epithelium. Exp. Eye Res..

[181] Esteve-Rudd J., Hazim R.A., Diemer T., Paniagua A.E., Volland S., Umapathy A., Williams D.S. (2018). Defective phagosome motility and degradation in cell nonautonomous RPE pathogenesis of a dominant macular degeneration. Proc. Natl. Acad. Sci. U. S. A..

[182] Dejos C., Kuny S., Han W.H., Capel H., Lemieux H., Sauve Y. (2018). Photoreceptor-induced RPE phagolysosomal maturation defects in Stargardt-like Maculopathy (STGD3). Sci. Rep..

[183] Lim J., Yue Z. (2015). Neuronal aggregates: Formation, clearance, and spreading. Dev. Cell.

[184] Forloni G., Terreni L., Bertani I., Fogliarino S., Invernizzi R., Assini A., Ribizzi G., Negro A., Calabrese E., Volonte M.A., Mariani C., Franceschi M., Tabaton M., Bertoli A. (2002). Protein misfolding in Alzheimer's and Parkinson's disease: Genetics and molecular mechanisms. Neurobiol. Aging.

[185] Schneiter R., Hitomi M., Ivessa A.S., Fasch E.V., Kohlwein S.D., Tartakoff A.M. (1996). A yeast acetyl coenzyme A carboxylase mutant links very-long-chain fatty acid synthesis to the structure and function of the nuclear membrane-pore complex. Mol. Cell Biol.

[186] Schneiter R., Kohlwein S.D. (1997). Organelle structure, function, and inheritance in yeast: A role for fatty acid synthesis?. Cell.

[187] Schneiter R., Tatzer V., Gogg G., Leitner E., Kohlwein S.D. (2000). Elo1p-dependent carboxy-terminal elongation of C14:1Delta(9) to C16:1Delta(11) fatty acids in Saccharomyces cerevisiae. J. Bacteriol..

[188] Bazan N.G. (2018). Docosanoids and elovanoids from omega-3 fatty acids are pro-homeostatic modulators of inflammatory responses, cell damage and neuroprotection. Mol. Aspects Med..

